# Sequential Self-Assembly of Polystyrene-*block*-Polydimethylsiloxane for 3D Nanopatterning *via* Solvent Annealing

**DOI:** 10.1021/acsami.4c08813

**Published:** 2024-07-22

**Authors:** Thanmayee Shastry, Jiayu Xie, Cheng-Hsun Tung, Teoh Yen Lynn, Aum Sagar Panda, An-Chang Shi, Rong-Ming Ho

**Affiliations:** † Department of Chemical Engineering, National Tsing Hua University, Hsinchu 30013, Taiwan; ‡ Department of Physics & Astronomy, McMaster University, Hamilton, Ontario L8S 4M1, Canada

**Keywords:** block copolymer, PS-*b*-PDMS, sequential self-assembly, layer-by-layer, solvent annealing, 3D nanopatterning

## Abstract

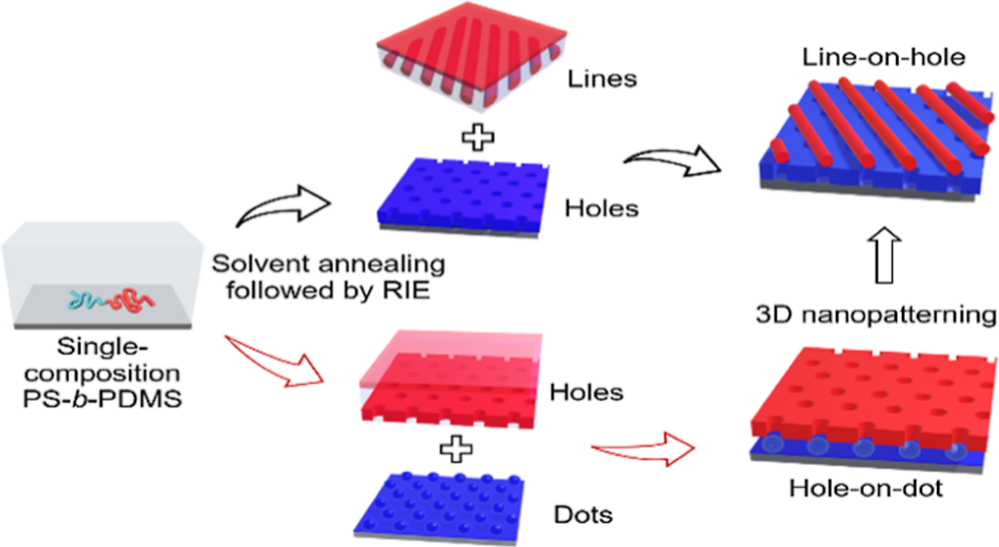

This study aims to develop a strategy for the fabrication of multilayer nanopatterns through sequential self-assembly of lamella-forming polystyrene-*block*-polydimethylsiloxane (PS-*b*-PDMS) block copolymer (BCP) from solvent annealing. By simply tuning the solvent selectivity, a variety of self-assembled BCP thin-film morphologies, including hexagonal perforated lamellae (HPL), parallel cylinders, and spheres, can be obtained from single-composition PS-*b*-PDMS. By taking advantage of reactive ion etching (RIE), topographic SiO_2_ monoliths with well-ordered arrays of hexagonally packed holes, parallel lines, and hexagonally packed dots can be formed. Subsequently, hole-on-dot and line-on-hole hierarchical textures can be created through a layer-by-layer process with RIE treatment as evidenced experimentally and confirmed theoretically. The results demonstrated the feasibility of creating three-dimensional (3D) nanopatterning from the sequential self-assembly of single-composition PS-*b*-PDMS *via* solvent annealing, providing an appealing process for nano-MEMS manufacturing based on BCP lithography.

## Introduction

Block copolymers (BCPs) have tremendous potential for applications in nanotechnologies due to their ability to microphase separate into well-ordered periodic nanostructures at sub-10 nm length scales.^1,2^ Specifically, varying the volume fraction of the blocks leads to the formation of a rich variety of ordered phase morphologies such as lamellae, cylinders, and spheres from BCPs.^3^ The self-assembled nanostructures of BCP thin films are utilized for several technological applications, such as nanolithography^1,2,4^ membranes,^5−7^ and optical films.^8−11^ Among different BCP systems, silicon-containing BCPs such as polystyrene-*block*-polydimethylsiloxane (PS-*b*-PDMS) provide a representative candidate for BCP lithography due to their high Flory–Huggins interaction parameter χ, which enables the formation of highly ordered sub-10 nm nanostructures coupled with an excellent etch contrast of one of the blocks of the BCP, making it feasible for pattern transfer.^12^ BCP thin films with well-ordered periodic nanostructures have attracted tremendous attention due to their potential in various practical applications. Solvent annealing is an effective approach to achieve highly regular BCP morphologies in the thin-film state due to the enhanced chain mobility in the presence of the solvent and the ability to kinetically capture self-assembled nanostructures upon rapid solvent evaporation.^13,14^ Furthermore, using a selective solvent for solvent annealing could create various morphologies from a single-composition BCP *via* the tuning of both volume fractions of the BCP and solvent selectivity.^15−17^ Additionally, the thin film morphology is dictated by several factors such as film thickness during solvent annealing and interfacial energy.^18,19^

As technology advances, intricate designs of three-dimensional (3D) nanostructures are essential for functional devices for applications in optics, microelectronics, *etc.*(20,21) For optical applications, it is necessary to justify the feature size of the self-assembled morphology, giving features on the order of the wavelength of light. However, the self-assembled nanostructures are nevertheless constrained by the intrinsic geometric features of the self-assembled morphologies of diblock copolymers.^19,22^ Therefore, enormous efforts have been devoted to developing strategies that could enrich geometric structures for the fabrication of 3D morphologies from BCP self-assembly processes.

Various strategies were developed to obtain novel 3D nanopatterning from layer-by-layer processes for 3D nanopatterning. Tavakkoli K. G. *et al.* demonstrated the formation of 3D multilevel nanostructures of cylindrical arrays with controllable angles, bends, and junctions from the self-assembly of BCPs on periodic arrays of posts.^23^ Additionally, 3D multilayered nanostructures could be fabricated through sequential stacking *via* cross-linking the underlying BCP layer, followed by subsequent BCP deposition process.^24−27^ Ruiz *et al.* proposed a technique by cross-linking the bottom cylindrical layer using UV light, followed by the deposition of the lamella-forming top BCP layer.^24^ Furthermore, Jung *et al.* demonstrated the formation of 3D nanostructures with lamellar/cylindrical multilayer using cross-linkable BCPs by a multilayer process.^26^ Also, 3D nanostructures were fabricated by directed or templated self-assembly from the immobilized underneath layer.^28−31^ Son *et al.* demonstrated such a technique where immobilized BCP patterns were used to direct the second BCP layer forming line-on-line, dots-on-line, and dots-in-hole hierarchical structures under different solvent annealing conditions.^28^ Rahman *et al.* extended this strategy to a responsive layering technique using sequential ordering and immobilization of BCP thin film to obtain a wide variety of 3D nanostructures *via* thermal annealing.^31^ Subsequently, Liu *et al.* showed the fabrication of metallic nanomesh using multimechanism of edge nucleation, trench wall guidance, and underlayer guidance.^32^

**Figure 1:**
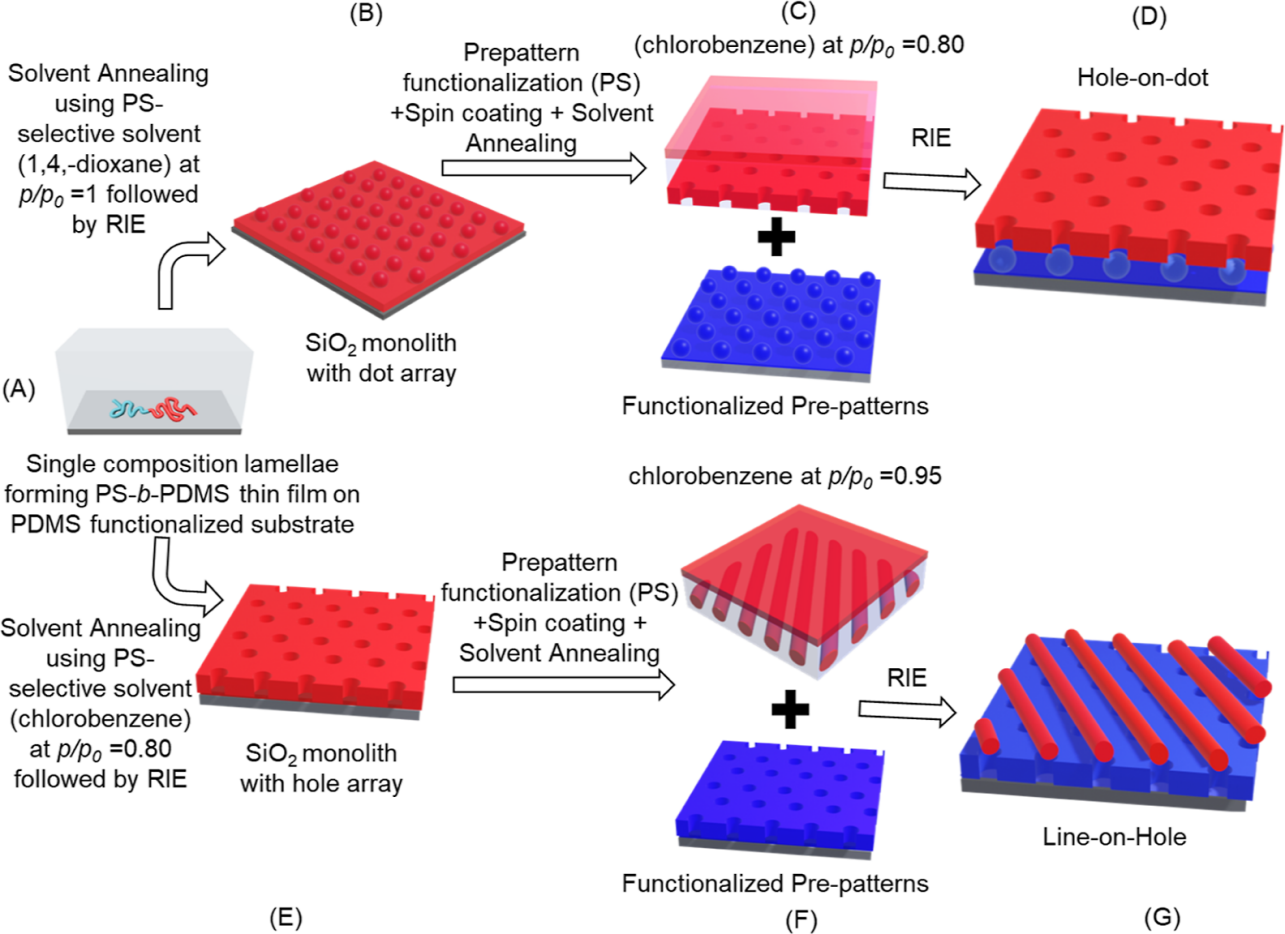
Schematic illustration of layer-by-layer sequential self-assembly. (A) Single-composition lamella-forming PS-*b*-PDMS thin film after spin-coating onto PDMS-functionalized substrate; (B) well-defined SiO_2_ monolith with a dot array obtained from solvent-annealed PS-*b*-PDMS thin film of (A) using 1,4 dioxane as a solvent at the relative vapor pressure of *p*/*p*_0_ = 1, followed by RIE; (C) functionalized prepattern obtained from (B) with PS brush, followed by solvent annealing using chlorobenzene as a solvent for solvent annealing at relative vapor pressure *p*/*p*_0_ = 0.80 to give the formation of HPL morphology in the thin-film state; and (D) hole-on-dot hierarchical texture after the RIE treatment of (C). (E) Well-defined SiO_2_ monolith with hole array obtained from solvent-annealed PS-*b*-PDMS thin film of (A) using chlorobenzene as a solvent at relative vapor pressure *p*/*p*_0_ = 0.80, followed by RIE. (F) Functionalized prepattern obtained from (E) with PS brush, followed by solvent annealing using chlorobenzene as a solvent at relative vapor pressure *p*/*p*_0_ = 0.95 to give the formation of parallel cylinders; (G) line-on-hole hierarchical texture after the RIE treatment of (F).

In the current work, we propose a simple methodology for the fabrication of 3D nanopatterning *via* solvent annealing of single-composition, lamella-forming PS-*b*-PDMS through sequential self-assembly. By taking advantage of the etching selectivity of PS-*b*-PDMS, a nanostructured thin film with various morphologies can be further treated with reactive ion etching (RIE) to produce topographic SiO_2_ patterns. Subsequently, the combination of the fabricated topographic monolith can be exploited to create a multilayer nanopattern through a layer-by-layer process. Our strategy is illustrated in Figure 1, which is a schematic showing sequential self-assembly using PS-*b*-PDMS with various PS-selective solvents for 3D nanopatterning. Specifically, lamella-forming PS-*b*-PDMS is spin-coated on a functionalized SiO_2_ layer with PDMS brushes using a neutral solvent (cyclohexane) to obtain a BCP thin film. With the use of PS-selective solvent (1,4 dioxane) at the relative vapor pressure of *p*/*p*_0_ = 1 for solvent annealing, the PS-*b*-PDMS forms a nanostructured monolith with hexagonally packed spheres. Note that the self-assembled morphologies in the thin-film state after solvent annealing with the use of PS-selective solvent give the formation of morphologies with PDMS as the minor phase. Owing to the low surface energy of PDMS, it is inevitable to give a thin wetting layer; as a result, the RIE treatment with the use of CF_4_/O_2_ etchant was first introduced to remove the PDMS wetting layer. Subsequently, an O_2_ etchant was used to convert the PDMS into SiO_2_ and remove PS simultaneously to give the SiO_2_ monolith with the aimed patterns. The conditions for the RIE treatments such as the power and time for the treatments will be dependent upon the self-assembled morphology in the thin-film state to give the aimed patterns. After RIE treatments, it produces a well-defined array of SiO_2_ dots (Figure 1B). The prepatterned array of dots will be further functionalized by using PS brushes, followed by spin-coating PS-*b*-PDMS thin film on the prepatterned first layer with SiO_2_ dots using cyclohexane for sequential self-assembly (Figure 1C). After solvent annealing using PS-selective solvent (chlorobenzene) at a relative vapor pressure of *p*/*p*_0_ = 0.80, a perforated layer [hexagonal perforated lamellae (HPL)] phase can be addressed onto the SiO_2_ dot array to give a hole-on-dot hierarchical texture after RIE treatments (Figure 1D). Following a similar approach, with the use of chlorobenzene at relative vapor pressure *p*/*p*_0_ = 0.80 for solvent annealing followed by RIE treatments on the first layer of the BCP thin film, the self-assembled PS-*b*-PDMS produces a SiO_2_ monolith with an array of hexagonal holes (Figure 1E). By directing the second-layer BCP thin film for solvent annealing with chlorobenzene at relative vapor pressure *p*/*p*_0_ = 0.95 on PS-functionalized prepattern SiO_2_ monolith, an array of parallel cylinders with a SiO_2_ line pattern can be formed aligned with the hole (Figure 1F). Consequently, as shown in Figure 1G, the resultant hierarchical texture exhibits a line-on-hole nanopattern after RIE treatments. Taken together, the procedure described here demonstrates the feasibility of using topographic SiO_2_ features as a template for sequential self-assembly for 3D nanopatterning.

## Experimental Section

### Material Synthesis

A linear PS-*b*-PDMS diblock copolymer, synthesized by sequential anionic polymerization with intrinsic properties [molecular weight (*M*) of PS and PDMS, *M*_n,PS_ = 47 kg/mol, *M*_n,PDMS_ = 33 kg/mol, polydispersity index = 1.05, and the volume fraction of PDMS = 0.46], was employed in this study. The synthesis of the PS-*b*-PDMS samples was accomplished by sequential anionic polymerization of styrene (Acros Organics, 99%) and hexamethylcyclotrisiloxane (D_3_, Acros Organics, 98%), employing high-vacuum techniques. Styrene was purified by distillation from CaH_2_ to dibutylmagnesium (Sigma-Aldrich, 1 M solution in heptane) and then stored into precalibrated ampules. Hexamethylcyclotrisiloxane (D_3_) was inserted in a round-bottom flask, diluted by an equal volume of purified benzene (C_6_H_6_), and stirred over CaH_2_. The solution (D_3_ and C_6_H_6_) was transferred into a flask containing polystyryllithium (PS^(−)^Li^(+)^) through a short path distillation apparatus by distillation of the solvent and sublimation of the D_3_, where it was stirred in the presence of PS^(−)^Li^(+)^ for approximately 2 h at room temperature, followed by sublimation and storage at precalibrated ampules. The solvents used for the polymerization were benzene (Riedel de Haen, 99.7%) and tetrahydrofuran (THF, Fisher Scientific, 99.99%). Benzene was purified *via* distillation from CaH_2_ and then stored under vacuum in the presence of (PS^(−)^Li^(+)^). Tetrahydrofuran was refluxed through metallic sodium and then distilled through CaH_2_ and Na/K alloy (3:1) under high-vacuum techniques and was stored under vacuum in calibrated ampules. The initiator used was *sec*-butyllithium (*sec*-BuLi, Sigma-Aldrich, 1.4 M in cyclohexane) after dilution in purified benzene, until the desired concentration was achieved. The termination reagents chosen were trimethylchlorosilane [ClSi(CH_3_)_3_, Sigma-Aldrich, 99+%] and tetrachlorosilane (SiCl_4_) (Aldrich, 99+%) that were accomplished through distillation from CaH_2_ and stored under high vacuum after several freeze-drying cycles. Methanol was used without any further purification for the precipitation but with a small quantity of antioxidant 2,6-di-*tert*-butyl-*p*-cresol (Aldrich, 99+%). Through this method, the PS-*b*-PDMS BCP sample was prepared.^37^ The *M* of PS and PDMS was determined using NMR and GPC. The transmission electron microscopy (TEM) (Figure S5a) and small-angle X-ray scattering (SAXS) (Figure S5b) results obtained after solution-casting of the PS-*b*-PDMS in bulk, obtained by using cyclohexane (a neutral solvent) followed by thermal annealing at 180 °C for 6 h, suggest that the sample was a lamella-forming BCP.

### Sample Preparation

The bulk sample of linear diblock PS-*b*-PDMS was prepared by a solution-casting method using a neutral solvent, cyclohexane, at room temperature. After drying the sample for 1 week with controlled evaporation, bulk samples were obtained, which were further dried in a vacuum oven for 3 days at 120 °C to eliminate the residual solvent. Subsequently, the bulk sample was thermally treated at 180 °C for 6 h with the use of differential scanning calorimetry and rapidly cooled to ambient conditions for TEM and SAXS experiments. To examine the thin-film morphology, PS-*b*-PDMS thin films with various thicknesses were prepared by spin-coating the BCPs using cyclohexane (neutral solvent) on a functionalized SiO_2_ substrate with PDMS brushes, followed by solvent annealing with different PS-selective solvent vapors with a controlled flow rate of solvent and nitrogen purge stream in a closed chamber equipped with spectral reflectometer for swelling thickness monitoring. To further examine the surface morphology of the PS-*b*-PDMS, a RIE treatment was carried out with mixed gases CF_4_ and O_2_, where CF_4_/O_2_ etchant was first used to remove the PDMS wetting layer, and then O_2_ etchant was used to convert the PDMS into SiO_2_ and remove PS simultaneously. Subsequently, the obtained topographic SiO_2_ prepatterns were functionalized with PS brushes followed by spin-coating of the second layer of BCP thin film onto the functionalized prepatterns to fabricate the hierarchical 3D double-layer nanopattern. The second layer of thin film was subjected to solvent annealing at respective annealing conditions, followed by the second step RIE process to generate different combinations of double-layer hierarchical nanopatterns with distinct topographic contrast.

### Instrumentation

TEM micrographs were obtained using JEOL-2100 TEM equipment with a 200 kV accelerating voltage. Swelling curves were obtained by using a spectral reflectometer (Filmetrics, F20-UV, wavelength range: 250–1500 nm). The spectrum was calibrated using a SiO_2_ layer silicon wafer. Field emission scanning electron microscopy was used to observe the morphological variations by using HITACHI SU8010 at a 10–12 kV accelerating voltage and with an 8 mm working distance. To avoid the charging problem, platinum with a thickness of approximately 2 nm was coated on the thin-film samples. The RIE was carried out on a Cello Technology model Nasca-20 instrument. Grazing-incidence SAXS (GISAXS) was carried out at the National Synchrotron Radiation Research Center (NSRRC), Taiwan. The samples were measured at a monochromatic beam energy of 10 kV, at a wavelength of 1.55 Å. The sample-to-detector distance was 4424.000 mm, and the grazing-incidence angle was 0.1°. The scattering data were obtained using a MAR165 CCD detector.

## Results and Discussion

### Controlled Self-Assembly of PS-*b*-PDMS on a Functionalized Substrate

**Figure 2:**
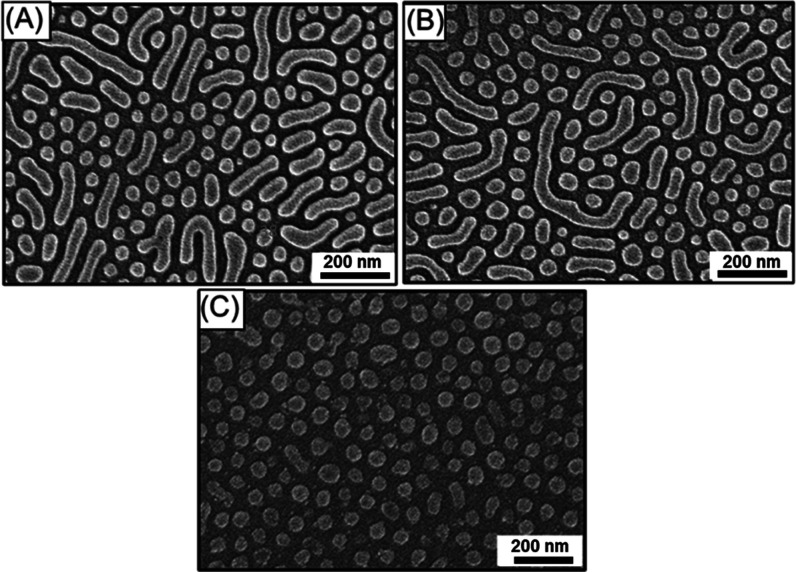
Top-view FESEM images of SiO_2_ monolith with dot array acquired when a BCP thin film with an initial film thickness of (A) ∼110, (B) ∼95, and (C) ∼65 nm is solvent annealed using 1,4-dioxane at *p*/*p*_0_ = 1.

Solvent annealing is used as a route to enrich the self-assembled morphologies of the BCPs in this work. The possibility of obtaining morphologies different from the equilibrium bulk phase of the neat BCPs *via* solvent annealing with selective solvent, especially in the thin-film state, has been previously demonstrated.^32^ For PS-*b*-PDMS with high χ, the film morphology is strongly dependent on the solvent selectivity, the annealing conditions (*e.g.* relative vapor pressure *p*/*p*_0_), and the initial film thickness. To reveal the effects of solvent and initial film thickness on self-assembled PS-*b*-PDMS thin film, thin films were spin-coated using cyclohexane solvent (neutral solvent) with different spin rates, resulting in thin films of different thicknesses of ∼65, ∼95, and ∼110 nm. Figure S6 shows the FESEM top-view images of the thin film morphologies prior to solvent annealing. The results suggest the presence of featureless morphology, indicating the poor ordered intrinsic lamella morphology. Subsequently, these samples of various initial film thicknesses were treated by solvent annealing using a PS-selective solvent. Note that the formation of various morphologies could be understood from the viewpoint of commensurability in terms of domain spacing. The commensurability effect is a phenomenon in which the ordered nanostructure can be formed at a certain ratio between the film thickness and the polymer domain spacing. The commensurability between the film thickness (*t*_0_) and polymer domain spacing (*d*_0_) can only be achieved when *t*_0_/*d*_0_ = *n*, where *n* = 1, 2, 3, ..., *N* for symmetric wetting conditions (if the same block wets both the air and substrate surface). With the use of a highly PS-selective solvent, 1,4-dioxane (solubility parameters, δ_PS_ = 9.1, δ_PDMS_ = 7.4, and δ_1,4-dioxane_ = 10.0 cal^1/2^ cm^–3/2^), for solvent annealing at a relative solvent vapor pressure of *p*/*p*_0_ = 1.00, the BCPs self-assembled into an array of spherical microdomains *via* solvent annealing. It is noted that the neat BCP is lamella-forming; thus, using a highly PS-selective solvent for solvent annealing leads to a great shift of the self-assembled structure from lamellae to spheres. As shown in Figure 2A,B, after solvent annealing followed by RIE, a mixed morphology of dots and lines was obtained, reflecting that there is a mismatch in the formation of a spherical array due to the incommensurability effect. By contrast, when the initial BCP thickness is lower, *e.g.*, ∼65 nm, an array of dots (Figure 2C) was successfully obtained after solvent annealing under a similar process condition, namely, an optimized thickness for the formation of a thin film with a spherical array was reached in this case.

**Figure 3:**
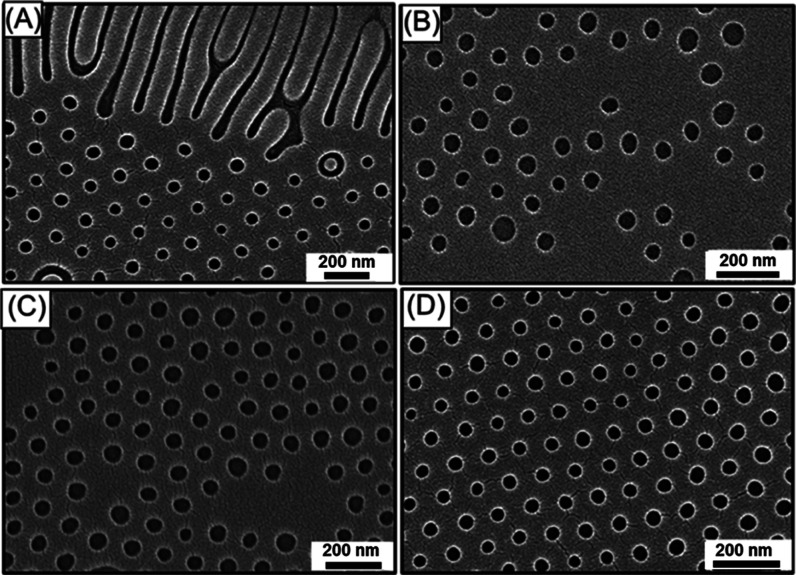
Top-view FESEM images of SiO_2_ monolith with hexagonal hole array acquired when a BCP thin film with an initial thickness of (A) ∼65, (B) ∼75, (C) ∼95, and (D) ∼110 nm is solvent annealed using chlorobenzene at *p*/*p*_0_ = 0.8.

Following a similar approach, by tuning the solvent selectivity to a moderately PS-selective solvent, chlorobenzene (solubility parameters, δ_chlorobenzene_ = 9.5 cal^1/2^ cm^3/2^), and the relative vapor pressure for solvent annealing, it is possible to manipulate the self-assembled motifs of the BCPs with different geometries, especially the targeted HPL. As shown in Figure 3A, after solvent annealing using chlorobenzene at *p*/*p*_0_ = 0.80 followed by RIE treatment, a porous texture was produced when the initial film thickness is ∼65 nm; yet, the porous textures include holes and a line pattern. The resultant textures might suggest the formation of mixed phases; intuitively, the formation of HPL and cylinders is based on the effective volume fraction for the PS segment. Interestingly, with increasing the initial film thickness to ∼75 nm under similar solvent annealing conditions, as shown in Figure 3B, the formation of an array of holes was observed; yet, the formed hole array was less regular. With a further increase in the initial film thickness to ∼95 nm (Figure 3C), a significant improvement of the ordering of the texture was observed. Most interestingly, once the initial film thickness was ∼110 nm, the formation of an array of holes with perfect hexagonal packing (Figure 3D) was produced. Under the thickness consideration, it is reasonable to infer that there is the formation of an HPL phase. As a result, the HPL phase appears in the BCP system under the conditions with an appropriate initial film thickness, implying that the film thickness indeed plays a prominent role in the formation of the self-assembled morphology under appropriate solvent annealing conditions. This result showed that it is possible to create a well-defined hole array with hexagonal lattice packing from the formation of HPL by solvent annealing the PS-*b*-PDMS thin film with an initial film thickness of ∼110 nm using PS-selective solvent at *p*/*p*_0_ = 0.80.

**Figure 4:**
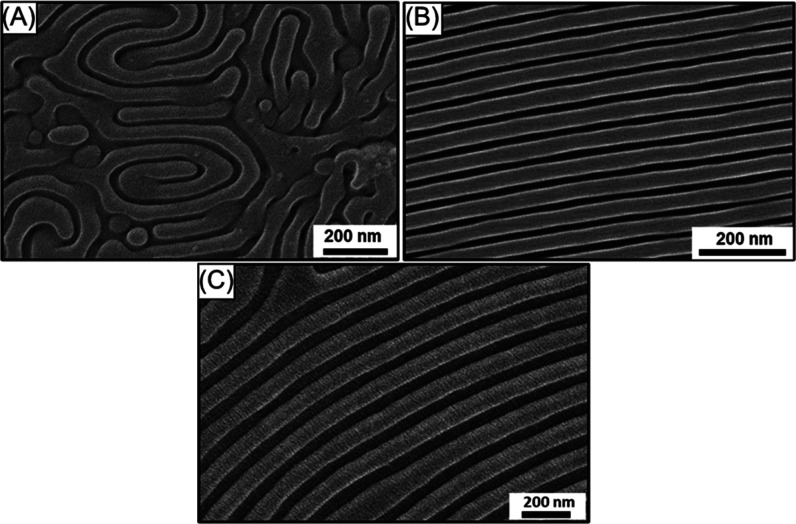
Top-view FESEM images of SiO_2_ monolith with line array acquired when a BCP thin film with an initial thickness of (A) ∼110, (B) ∼95, and (C) ∼65 nm is solvent annealed using chlorobenzene at *p*/*p*_0_ = 0.95.

Upon raising the relative vapor pressure of the same moderately PS-selective solvent (chlorobenzene) to *p*/*p*_0_ = 0.95, the formation of parallel cylinders was observed. Note that along with the solvent selectivity, the degree of swelling could be reasonably tuned by the *p*/*p*_0_ value, which leads to the formation of the cylinder phase. The segmental interaction between PS and PDMS after swelling can be quantified by using an effective Flory–Huggins interaction parameter, χ_eff_, which correlates with the volume fraction of solvent, ϕ_s_, or swelling ratio (SR) as shown in eq 1.
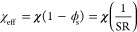
1Accordingly, the variation in SR due to the change in the relative solvent vapor pressure (*p*/*p*_0_) may subsequently alter the effective volume fraction of PDMS (*f*_PDMS_^eff^) as formulated in eq 2, resulting in the compositional asymmetry of phase behavior due to swelling.
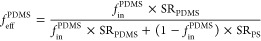
2where, *f*_in_^PDMS^ is the intrinsic volume fraction of PDMS, SR_PDMS_ is the SR of PDMS homopolymer, and SR_PS_ is the SR of PS homopolymer. Accordingly, at *p*/*p*_0_ = 0.80, the formation of perforated lamellae can be achieved at SR of 1.33, as shown in Table 1, with the volume fraction of solvent (ϕ_s_) of 0.25 in the BCP microdomain, giving the reduction of *f*_PDMS_^eff^ from 0.46 to 0.41, thus driving the phase transformation from intrinsic lamellae toward perforated lamellae. In contrast, once the *p*/*p*_0_ reaches 0.95, with a higher SR of 1.73, giving ϕ_s_ of 0.42 in the BCP. As shown in Figure 4A, when the initial BCP thin film thickness of ∼110 nm is subjected to solvent annealing using chlorobenzene at *p*/*p*_0_ = 0.95, parallel cylinders are formed; yet, the formed line array is nonuniform with several line bending. By contrast, with the reduction of *f*_PDMS_^eff^ from 0.41 to 0.34, well-aligned parallel cylinders are observed when the initial BCP film thickness is reduced to ∼95 nm (Figure 4B) and ∼65 nm (Figure 4C) for the solvent annealing with chlorobenzene at *p*/*p*_0_ = 0.95. However, the cylinders formed with an initial film thickness of ∼65 nm are susceptible to some dislocation defects; thus, the parallel cylinders formed from an initial BCP film thickness of ∼95 nm can be considered the optimized condition for the formation of the line array. The formation of the resulting phases and the corresponding variables is summarized in Table 2.

**Table 1:** Solvent Annealing Parameters of PS-*b*-PDMS Using Chlorobenzene and 1,4-Dioxane

solvent	chlorobenzene		1,4-dioxane
*p*/*p*_0_	0.80	0.95	1.00
SR	1.33	1.73	2.33
ϕ_s_	0.25	0.42	0.57
*f*_PDMS_^eff^	0.41	0.34	0.20

**Table 2:** Resultant Phases and Their Corresponding Optimum Conditions Needed for the Formation of Morphologies

morphology	initial film thickness (nm)	solvent used	relative vapor pressure (*p*/*p*_0_)
sphere (dot array)	∼65	1,4-dioxane	1
HPL (hole array)	∼110	chlorobenzene	0.8
parallel cylinders (line array)	∼95	chlorobenzene	0.95

**Figure 5:**
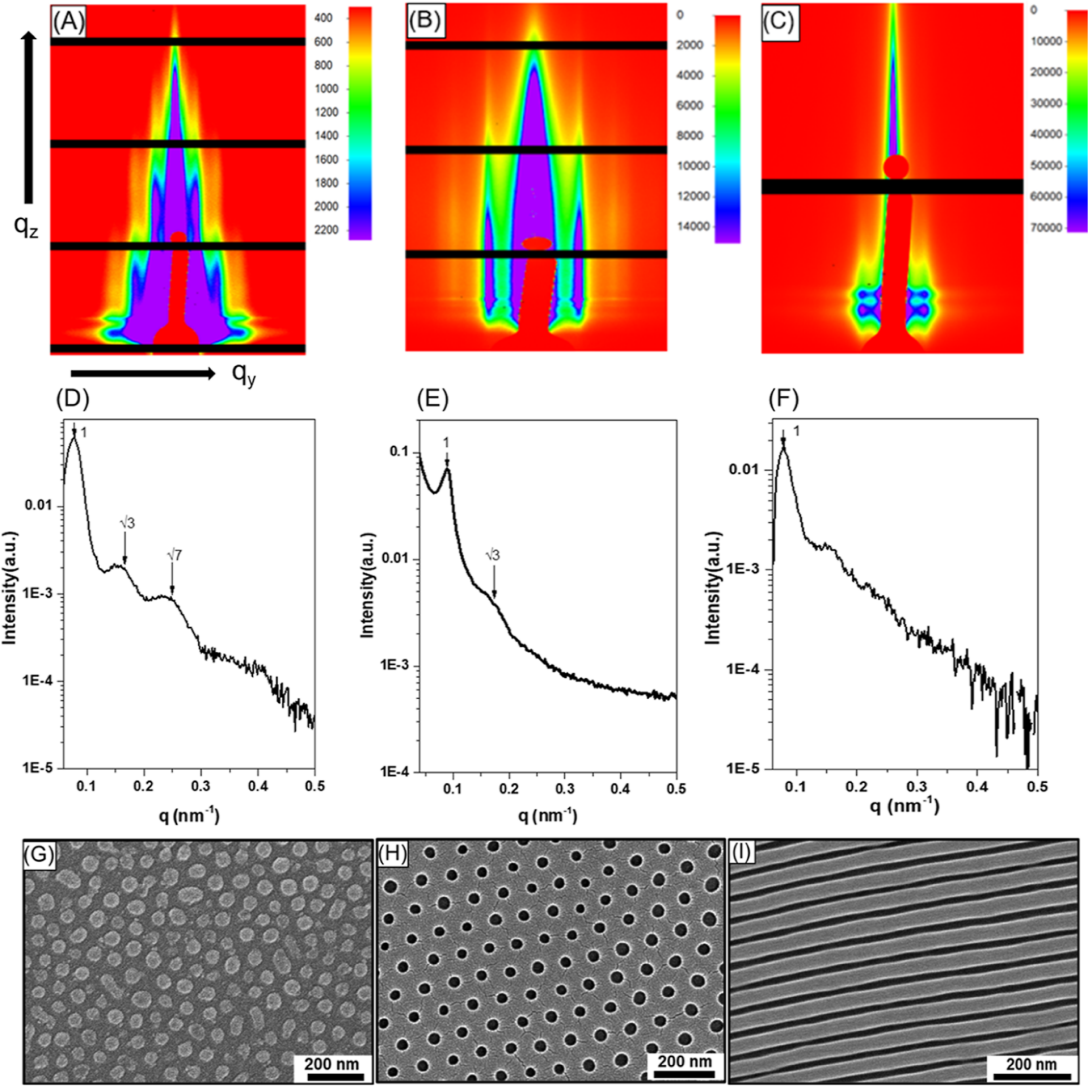
2D GISAXS pattern of PS-*b*-PDMS thin film with (A) sphere, (B) HPL, and (C) cylinder morphologies, respectively. The corresponding 1D line profile of PS-*b*-PDMS thin film with (D) sphere, (E) HPL, and (F) parallel cylinder morphologies, respectively. Top-view FESEM images of SiO_2_ monolith with (G) dot array, (H) hexagonal hole array, and (I) line array fabricated.

Furthermore, GISAXS was used to confirm the self-assembled morphology in the thin-film state (Figure 5A–F). Figure 5A shows the two-dimensional (2D) GISAXS pattern of PS-*b*-PDMS thin film with an initial film thickness of ∼65 nm subjected to solvent annealing using 1,4-dioxane as a solvent at the relative vapor pressure of *p*/*p*_0_ = 1, and Figure 5D shows the corresponding one-dimensional (1D) GISAXS line profile (*q vs* intensity); the reflection peaks occur at the relative *q* values of 1:√3:√7. By combining FESEM observation (Figure 5G) for real-spacing imaging, the scattering results from reciprocal-space imaging suggest the formation of a sphere morphology with hexagonal packing in the thin-film state. Also, the results suggest that the 2D GISAXS pattern appears similar to the results obtained by Korgel and co-workers for the spheres.^34^

Similarly, Figure 5B shows the 2D GISAXS pattern of PS-*b*-PDMS thin film with an initial film thickness of ∼110 nm subjected to solvent annealing using chlorobenzene as a solvent at the relative vapor pressure of *p*/*p*_0_ = 0.8, and Figure 5E shows the corresponding 1D GISAXS line profile (*q vs* intensity); the reflection peaks occur at the relative *q* values of 1:√3. By combining FESEM observation (Figure 5H) for real-spacing imaging, the scattering results from reciprocal-space imaging suggest the formation of the HPL morphology with hexagonal packing in the thin-film state. Note that the 2D GISAXS pattern appears similar to the results obtained by Yager and co-workers for the HPL morphology.^35^ Also, Figure S7 shows the cross-sectional FESEM image from the HPL monolayer examined by using FIB for sectioning of the RIE-treated sample. Note that the sample was coated with platinum (Pt) coating as a protective layer to avoid the charging effect. The Pt-coated sample acts as a guiding line to visualize the forming hole array, and it appears as an alternating concave and protruding structure, with the concave region corresponding to the PS perforation after degeneration while the protruding region refers to the converted SiO_2_ microdomain from PDMS. As a result, the forming morphology was identified as HPL by the GISAXS as well as top-view and cross-sectional FESEM images. Furthermore, Figure 5C shows the 2D GISAXS pattern of PS-*b*-PDMS thin film with an initial film thickness of ∼95 nm subjected to solvent annealing using chlorobenzene as a solvent at the relative vapor pressure of *p*/*p*_0_ = 0.8, and Figure 5F shows the corresponding 1D GISAXS line profile (*q vs* intensity). By combining FESEM observation (Figure 5I) for real-spacing imaging, the scattering results from reciprocal-space imaging suggest the formation of a parallel cylinder morphology in the thin-film state. We speculate that the Bragg peak in the *z*-direction of the 2D GISAXS pattern suggests the presence of parallel cylinder morphology.

The self-assembled morphologies and the corresponding ordering strongly depend on the film thickness due to the commensuration effect. The formation of various morphologies could be understood from the viewpoint of commensurability, in terms of domain spacing. Note that in the case of solvent annealing, the swollen film thickness determines the commensurability with the microdomain spacing.^33^ Also, the commensurability between the film thickness and polymer domain spacing can be achieved when *t*_0_/*d*_0_ = *n* (*n* = 1, 2, 3, ...).^36^ Thus, we anticipate that the swollen film thickness should be commensurate with the BCP microdomain spacing. The microdomain spacing of each morphology was calculated from GISAXS measurements. As evidenced by the 1D GISAXS profile, the *d*-spacing of sphere morphology (*d*_sphere_) was determined as ∼76 nm, and for the formation of BCP thin film with sphere array, the SR was determined to be 2.33 (Table 1.), giving the swollen thickness around ∼151 nm and the ratio of *t*_swell_/*d*_sphere_ = ∼2. Thus, when the initial thickness of BCP thin film is ∼60 nm, the swollen film thickness is commensurate with the BCP microdomain period, giving rise to the formation of spherical morphology. Similarly, the *d*-spacing of HPL (*d*_HPL_) from 1D GISAXS profile was calculated as ∼73 nm, and the SR achieved by solvent annealing the thin film with chlorobenzene at *p*/*p*_0_ = 0.80 is 1.33, giving the swollen film thickness ∼147 nm and the ratio of *t*_swell_/*d*_HPL_ = ∼2. Thus, with the initial thickness of BCP thin film being ∼110 nm, the swollen film thickness is commensurate with the BCP microdomain period, leading to the formation of HPL morphology. Furthermore, the *d*-spacing of cylinder (*d*_cylinder_) from 1D GISAXS profile was determined as ∼78 nm, and the SR achieved by solvent annealing the thin film with chlorobenzene at *p*/*p*_0_ = 0.80 is 1.73 (Table 1.), giving the swollen film thickness ∼164 nm and the ratio of *t*_swell_/*d*_cylinder_ = ∼2. Thus, with the initial thickness of BCP thin film being ∼95 nm, the swollen film thickness is commensurate with the BCP microdomain period, giving rise to the formation of parallel cylinder morphology. Therefore, by tuning the solvent selectivity, film thickness, and optimized conditions for solvent annealing, it is possible to use 3D nanopatterning to give various SiO_2_ monoliths with different ordered textures. Consequently, a dot array can be acquired when a BCP thin film with the initial thickness of ∼65 nm was solvent annealed using a 1,4-dioxane at *p*/*p*_0_ = 1.00; hole array is achieved when a ∼110 nm thin film was solvent annealed using chlorobenzene at *p*/*p*_0_ = 0.8; and finally, a line array is acquired when a ∼95 nm thin film was solvent annealed using chlorobenzene at *p*/*p*_0_ = 0.95, followed by a RIE treatment. Based on these results, it is reasonable to conclude that there are several important factors for the controlled self-assembly of the PS-*b*-PDMS thin film at which the solvent selectivity will be the primary factor to give rise to the formation of aimed self-assembled morphologies due to the consideration of the effective volume fraction. With the increase of PS-selectivity for solvent annealing, it is possible to create composition asymmetry including sphere, HPL, and cylinder. For the better ordering of the aimed morphology, it is necessary to tune the thickness to fulfill the commensuration condition. Moreover, as demonstrated above, it is also possible to control the solvent annealing condition by tuning the relative vapor pressure, giving the fine-tuning of the effective volume fraction.

### Fabrication of Layer-by-Layer Nanopatterns

**Figure 6:**
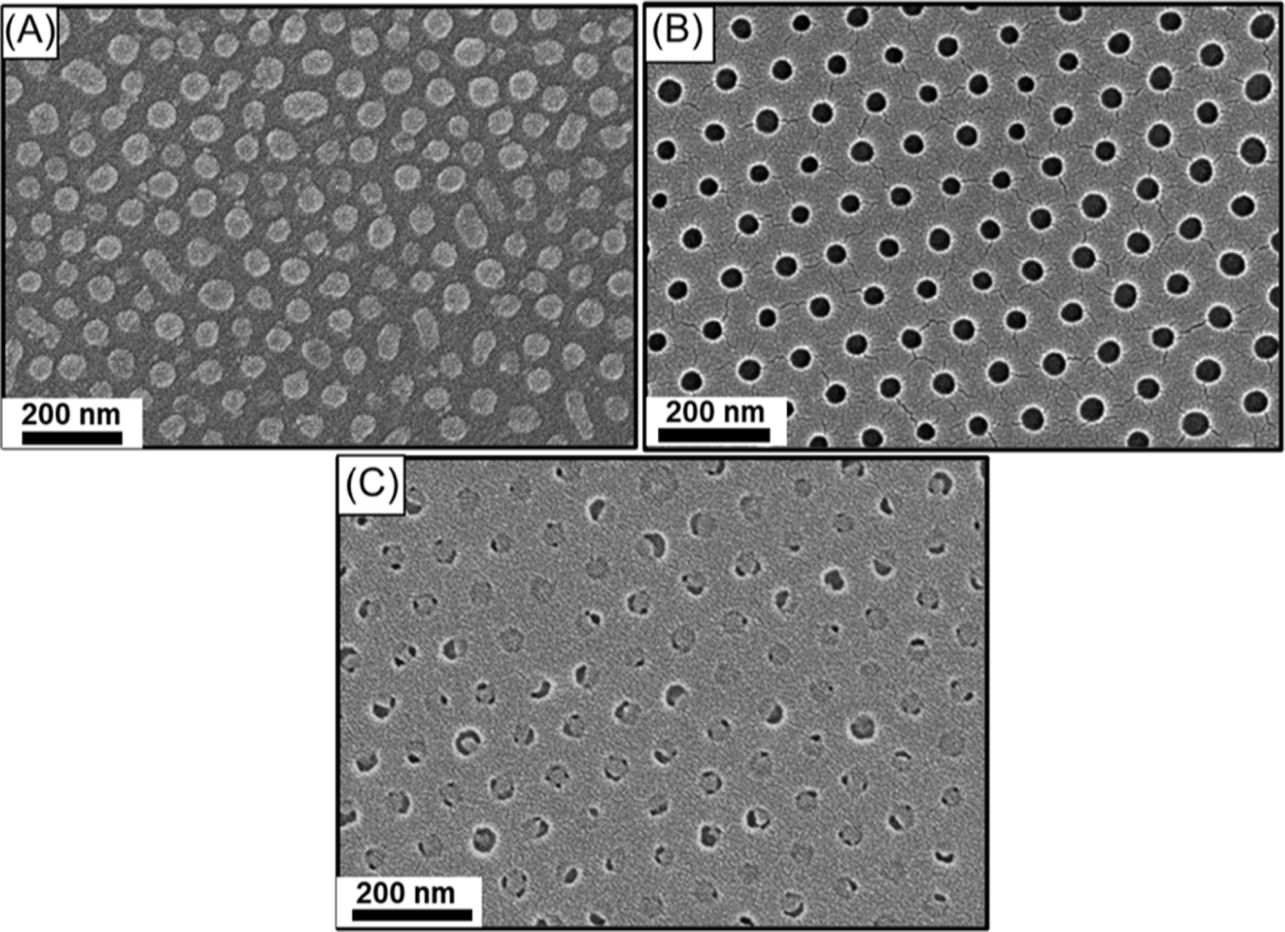
Top-view SEM images of (A) SiO_2_ monolith with dot array, (B) SiO_2_ hexagonal hole, and (C) hole-on-dot nanopattern.

**Figure 7:**
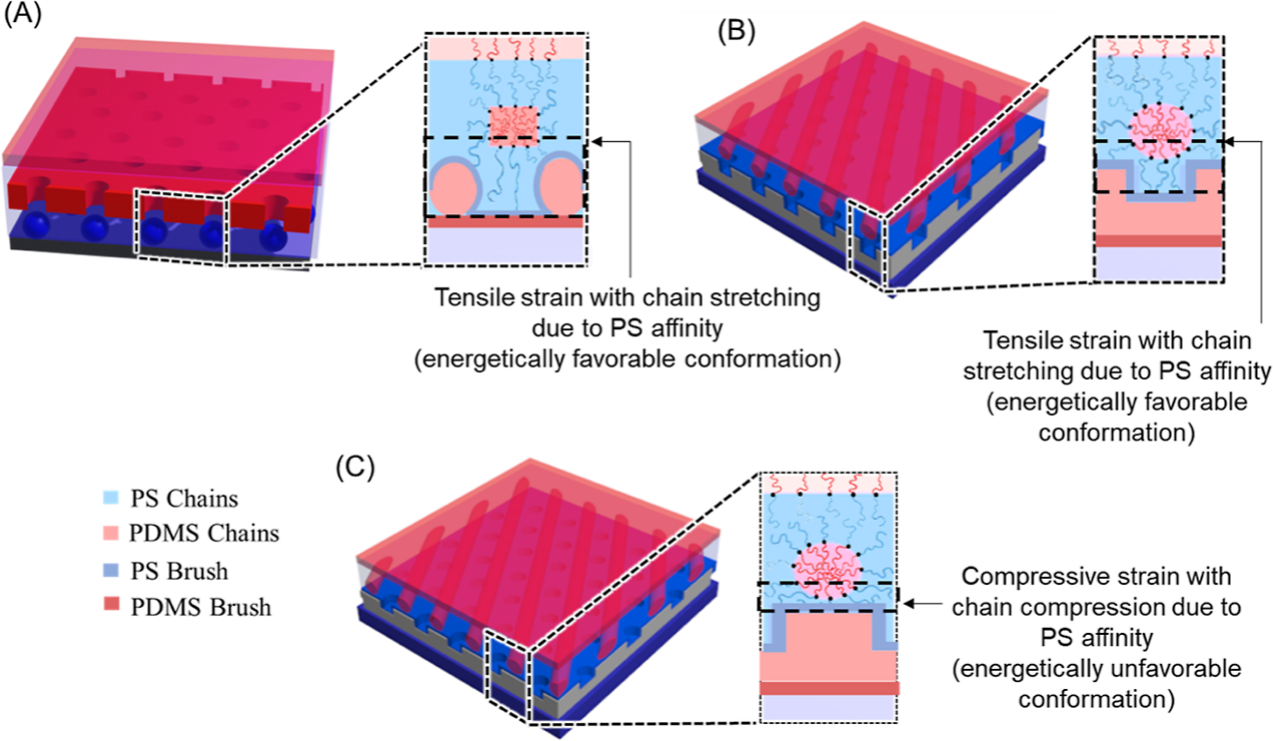
Schematic illustration of layer-by-layer sequential self-assembly for the fabrication of (A) hole-on dot and (B,C) line-on-hole nanopattern.

In this subsection, we demonstrate a strategy of using hierarchical self-assembly to create a 3D nanopatterned thin film by using a layer-by-layer sequential process. The formation of the hole-on dot could be realized when the first layer is composed of topographic SiO_2_ monolith with a dot array that was fabricated by solvent annealing a BCP thin film with the initial film thickness of ∼65 nm using a 1,4-dioxane at relative solvent vapor pressure of *p*/*p*_0_ = 1.00, followed by RIE treatment (Figure 6A). Subsequently, the prepatterned monolith was grafted by PS–OH brush and then addressed with the second layer obtained by solvent annealing with chlorobenzene at *p*/*p*_0_ = 0.80 on the PS-functionalized SiO_2_ monolith fabricated above; a well-ordered hole array with hexagonal packing can be formed in the second layer (Figure 6B). The reason to functionalize the SiO_2_ monolith with a sphere array is to create an HPL morphology with no PDMS wetting layer from the substrate; with that, it is possible to carry out the RIE treatments for the aimed hole-on-dot texture to avoid the formation of multilayer PDMS underneath the PDMS wetting layer that will cause difficulty in creating the holes as expected unless a delicate RIE cycle with different etchants is finely conducted. Consequently, as shown in Figure 6C, the resultant double-layer nanopattern exhibits the hole-on-dot nanopattern after the second RIE treatment, indicating the feasibility of using the underneath topographic SiO_2_ monolith as a template for the controlled self-assembly of the second layer PS-*b*-PDMS through solvent annealing. To give a better insight into the formation of the hole-on dot nanopattern, the molecular dispositions for the self-assembled PS-*b*-PDMS hole array should be aligned along the underlying dot patterns to achieve the most stable state in thermodynamics. Such a scenario is illustrated in Figure 7A for the molecular dispositions of polymer chains with the topographical SiO_2_ monolith. While the array of hole nanostructures is aligned by the dot array, the polymer chains will be able to render sufficient space for the uniform chain disposition within the dot, thus giving the most energetically favorable conformation. Confined BCP arrays can exhibit tensile or compressive strain, and the tension is easier to accommodate than compression, in line with the previously reported results.^41^ Also, the free-energy change associated with the straining of the BCP lattice can be approximated using a model that considers the impact of strain on both the conformational entropy of a polymer chain and the interfacial energy between the BCP domains. As a result, the HPL nanostructures tend to be aligned by the dot array of the underlying SiO_2_ monolith to attain the best stability.

Additionally, the well-aligned hole onto the underlying dot can be achieved, attributed to the grafting of the PS–OH brush layer onto the SiO_2_ monolith with a dot array before the spin-coating of second layer for solvent annealing. The presence of the PS–OH brush layer should be carried out for the modification of the affinity of PS-*b*-PDMS with the underneath layer to acquire the aimed HPL texture as demonstrated. Also, it is interesting to realize it is feasible to justify the self-assembled HPL with better matching for the *d*-spacing to give the aimed hole-on-sphere array *via* the positioning of the self-assembled perforated PS microdomain (that will be the hole nanostructures after RIE treatment) into the underlying spheres. It may suggest that there is a capability to adjust the required spacing matching due to the malleable character of the BCP. To approximately calculate the domain spacing of the HPL and hexagonally packed spheres after RIE, ImageJ analysis was utilized. By a quantitative analysis of Figure 6A,B, the domain spacing of the sphere can be calculated as ∼40 nm and that of HPL is ∼43 nm. Thus, it might be possible to accommodate a hole on top of a sphere. However, it is possible to observe certain defects that can be attributed to defects present in the first layer.

**Figure 8:**
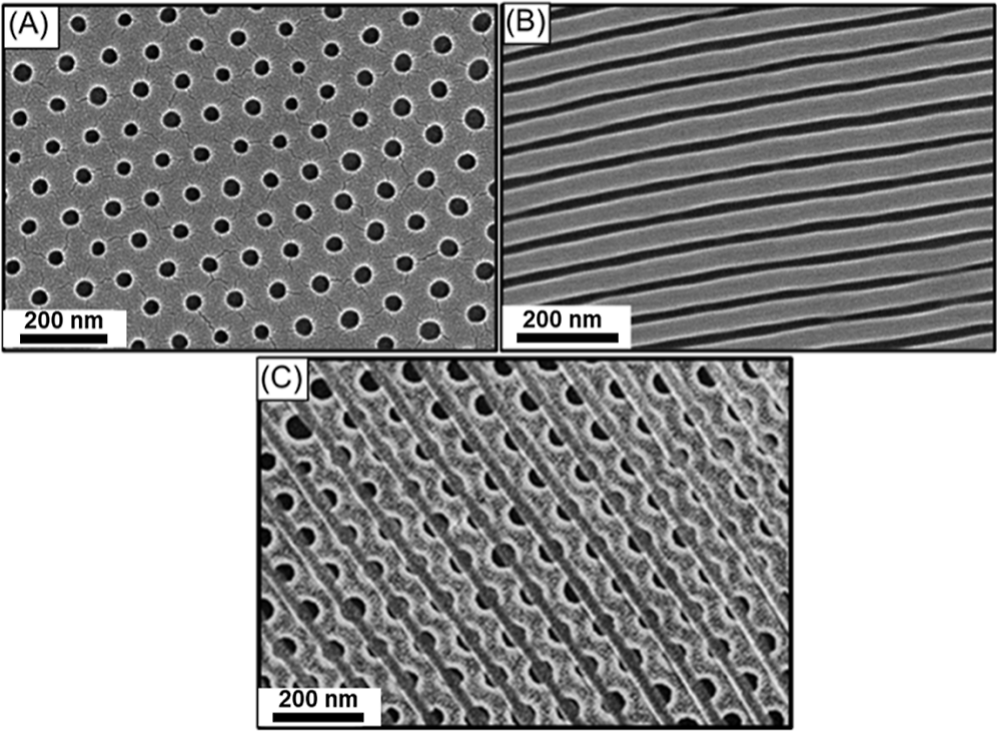
Top-view SEM images of (A) SiO_2_ monolith with a hexagonal hole array, (B) SiO_2_ line array, and (C) line-on-hole nanopatterns fabricated.

To further demonstrate the feasibility of the aimed 3D nanopatterning, the formation of a topographic SiO_2_ hole pattern was fabricated by using the HPL array as a first layer obtained from solvent annealing PS-*b*-PDMS thin film using chlorobenzene at *p*/*p*_0_ = 0.80, followed by RIE treatment (Figure 8A); subsequently, the SiO_2_ hole template was grafted by PS–OH brush and then used to address the forming parallel cylinders from solvent annealing as the second layer obtained by solvent annealing the BCP thin film with chlorobenzene at *p*/*p*_0_ = 0.95 (Figure 8B). By addressing the parallel cylinder array on the functionalized SiO_2_ hole template, it gives rise to a line-on-hole nanopattern, as shown in Figure 8C, with one-to-one accommodation after the second RIE treatment. Thus, it further evidences the feasibility of driving the controlled self-assembly of the second layer PS-*b*-PDMS onto the underneath topographic SiO_2_ monolith through solvent annealing with functionalization of the topographical template.

Furthermore, from the viewpoint of molecular chain dispositions for thermodynamically favored states, the line nanostructures must be positioned on the underlying hole to relieve the chain stretching and compression, and when the cylinder nanostructures are aligned by the hole array, the polymer chains will be able to render sufficient space for the uniform chain disposition within the hole, giving the most energetically stable conformation (Figure 7B). By contrast, while the cylinder nanostructures are aligned on the bulge region, the polymer chains near the bottom side will experience significant chain stretching and compression to fit in the crowded region, which is energetically unfavorable (Figure 7C). Therefore, it is reasonable that the cylinder nanostructures tend to be aligned by the hole array of the underlying SiO_2_ monolith to attain the best stability. Additionally, the unidirectional well-aligned parallel cylinders onto the underlying hole can be achieved, attributed to the grafting of the PS–OH brush layer onto the SiO_2_ monolith with a hexagonal hole array before the spin-coating of the second layer for solvent annealing. The presence of a PS–OH brush layer may facilitate the modification of the affinity of PS-*b*-PDMS in the top layer with respect to the bottom layer by changing the wetting conditions. As a result, the PS homopolymer may tailor the interaction with the major PS microdomain in the top layer. Furthermore, the directional alignment of cylinders on the hole array can be depicted from the viewpoint of commensurability in terms of domain spacing matching between the top layer and the underneath layer. By ImageJ analysis of Figure 8A,B, the domain spacing of HPL can be calculated as ∼43 nm and that of cylinders is ∼37 nm. As evident from Figure 8C, a certain degree of contraction of the second layer is essential for accommodation of the line on the hole. Thus, it might be possible to accommodate a line on the top of the hole.

### SCFT Results for 3D Nanopatterning

**Figure 9:**
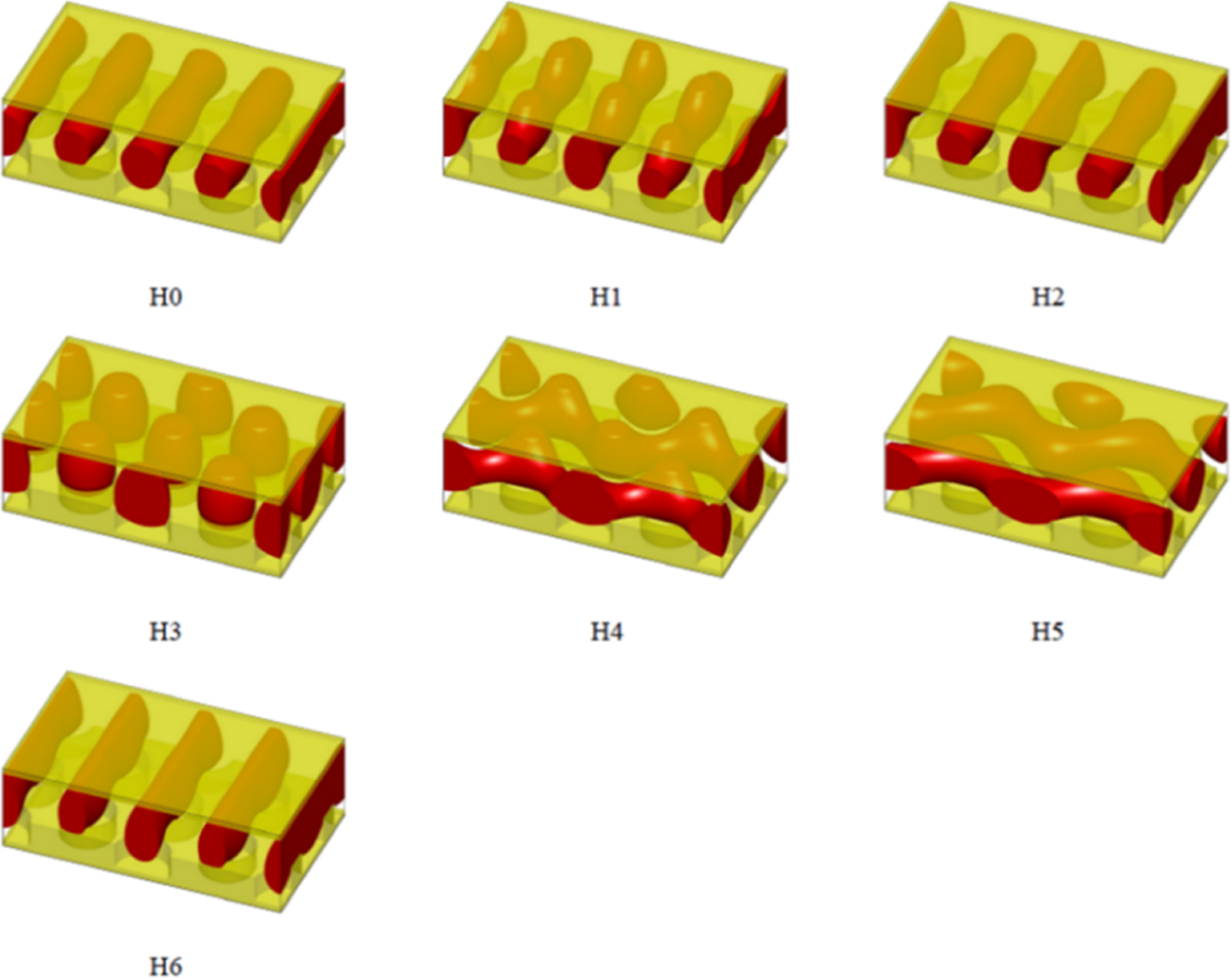
Discovered nanostructures with relatively low free energies when the bottom wall geometry is holes. These phases are used in calculating the free energy curves in Figure 11A.

**Figure 10:**
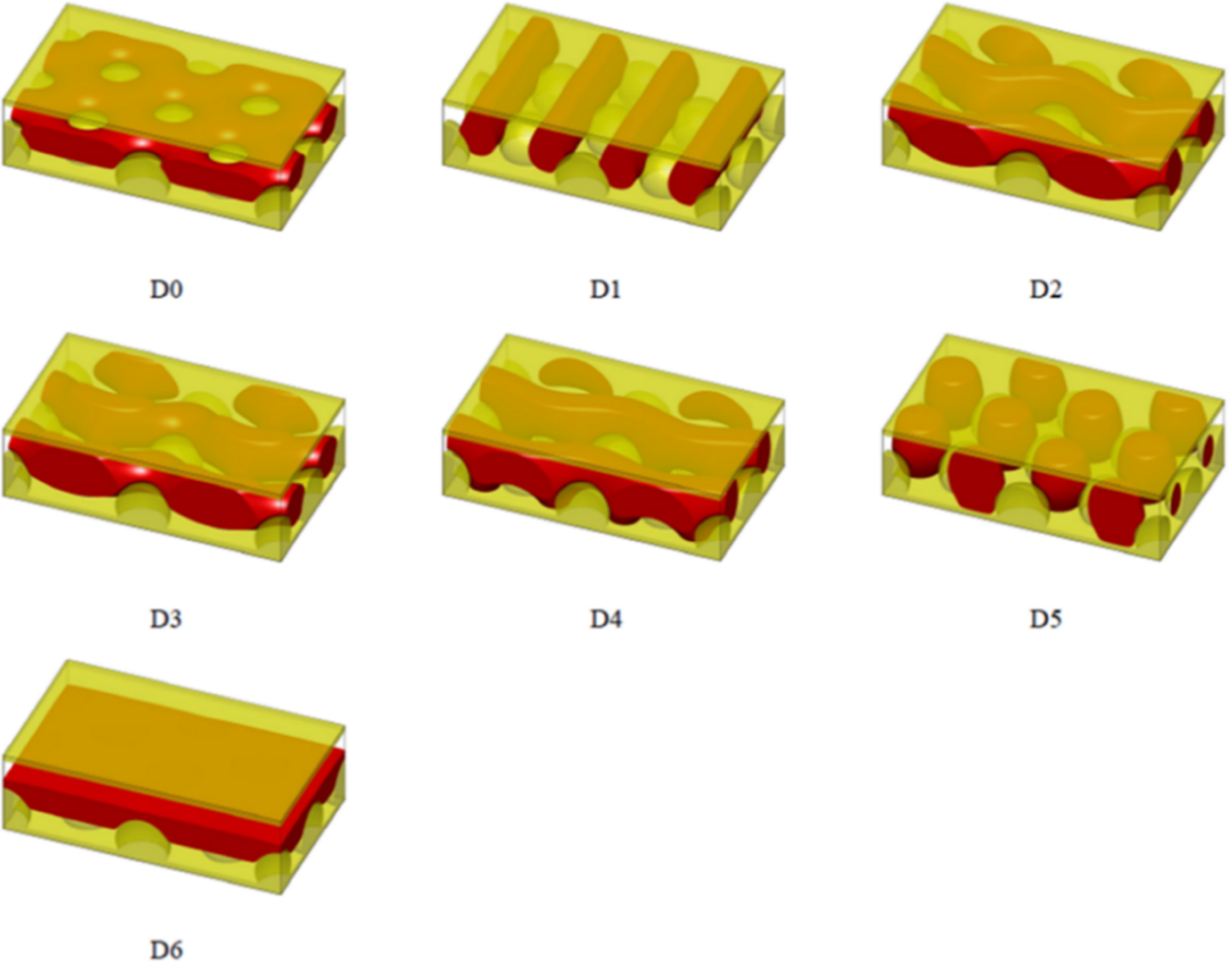
Discovered nanostructures with relatively low free energies when the bottom wall geometry is dots. These phases are used in calculating the free energy curves in Figure 11B.

Self-consistent field theory (SCFT) calculations were carried out to understand and confirm the feasibility of fabricating 3D nanopatterns such as hole-on-dot and line-on-hole (see Supporting Information for details). The candidate structures considered in our SCFT calculations are generated using two methods. In the first method, a random value between 0 and 1 is drawn from a uniform distribution and assigned to ϕ_A_(***r***) at each spatial point, while the value of ϕ_B_(***r***) is calculated by using the incompressibility condition, *i.e.*, ϕ_B_(***r***) = 1 – ϕ_W_(***r***) – ϕ_A_(***r***). We then iterate the SCFT equations to equilibrate the system, with a maximum number of iterations set to 10,000. For each bottom-wall geometry, this procedure is carried out 300 times with small, medium, and large film thicknesses *D*. All the converged structures obtained from this procedure are used as the candidate phases for further calculations. In the second method, we follow a similar procedure but initialize the polymer densities by placing spheres, cylinders, lamellae, or perforated lamellae with different spacings and orientations as the initial profile for ϕ_A_(***r***). Then, the incompressibility condition is used to initialize ϕ_B_(***r***). Similarly, these morphologies are equilibrated by SCFT, and all of the converged structures are included as our candidate phases. Next, we perform SCFT calculations for all candidate structures discovered *via* the above two approaches over a range of *D*. For holes and dots in bottom-wall geometries, *D* spans from 2.5*R*_g_ to 3.5*R*_g_ and from 3*R*_g_ to 4*R*_g_, respectively, both with increments of 0.1*R*_g_. During our calculations, we discovered over a dozen distinct structures for both the hole and dot geometries. Figures 9 and 10 show the schematics of structures with relatively lower free energies under the hole and dot geometries, respectively. Structures with significantly higher free energies are omitted in both Figures 9 and 10 for brevity. In Figure 9, several structures exhibit a line-on-dot pattern, reminiscent of that observed in experiments, including H0, H1, H2, and H6. In Figure 10, only D0 exhibits a hole-on-dot pattern. Among all the nanostructures, random initialization only converged to H0 and D0. Specifically, when starting with randomly initialized polymer densities, the SCFT equations converged to the H0 structures 33 out of 300 times under the hole geometry, while the equations converged to the D0 structure 200 out of 300 times under the dot geometry. The rest of the runs did not converge. All the other structures were discovered exclusively by using the second method. This indicates that H0 and D0 are likely the stable nanostructures when the bottom-wall geometries are holes and dots, respectively.

**Figure 11:**
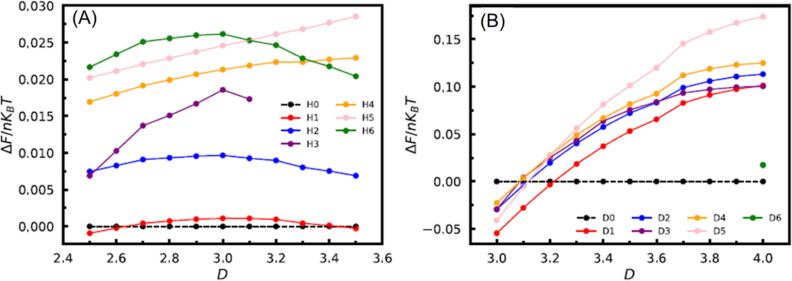
Free energies per chain of structures relative to that of the (A) H0 structure when the bottom wall geometry is holes and the (B) D0 structure when the bottom wall geometry is dots.

More explicit evidence is provided by comparing the free energies of the different structures. In Figure 11A, the free energies per chain of various structures (H0–H6) under the hole geometry are shown relative to that of the H0 structure, as a function of *D*. It is observed that the H0 and H1 structures have comparable free energies, which are significantly lower than those of the other structures across the entire range of *D* from 2.5*R*_g_ to 3.5*R*_g_. Both the H0 and H1 structures exhibit a line-on-hole pattern, consistent with the experimental observations. Figure 11B shows that the D0 structure, conforming to the hole-on-dot pattern, has the lowest free energy over a large range of *D*, *i.e.*, from ∼3.21*R*_g_ to 4*R*_g_. When *D* ≲ 3.21*R*_g_, the D1 structure becomes more stable. Figure S4 provides more detailed visualizations of the H0, H1, and D0 structures from different perspectives. Notably, the H0 and H1 structures exhibit very similar morphological characteristics. The main distinction between these two structures is that the cylinders in the H0 structure have more flat surfaces, while those in the H1 structure have more curved surfaces.

These SCFT results suggest that within a wide range of film thickness *D*, the line-on-hole and hole-on-dot patterns represent thermodynamically favorable arrangements for the self-assembly of AB diblock copolymers. These theoretical results are in qualitative agreement with our experiments, providing theoretical evidence for the experimentally observed layer-by-layer nanopatterns. The theoretical studies further demonstrated the feasibility of fabricating 3D nanopatterns such as hole-on-dot and line-on-hole using single-composition lamellae forming a PS-*b*-PDMS thin film under solvent annealing conditions. Note that the formation of the 3D nanostructures such as hole-on-dot and line-on-hole occurs due to the chemical effect combined with the topographical effect from guiding first layer, in line with previous studies reported.^38−41^ This can be further evidenced by SCFT calculations at which the hole aligned with dot bottom geometry, and the line aligned with hole bottom geometry has the minimum free energies per chain while the bottom wall has an affinity toward one of the constituted blocks. As a result, the formation of hole-on-dot and line-on-hole nanostructures can be driven by the bottom wall geometry (dot or hole) with chemical affinity toward a specific block.

## Conclusions

In conclusion, the phase behaviors of PS-*b*-PDMS can be regulated by solvent annealing of a single-composition, lamella-forming PS-*b*-PDMS, giving rise to various self-assembled morphologies such as perforated lamellae (HPL), parallel cylinders, and spheres under appropriate annealing conditions related to the selection of PS-selective solvent, the relative vapor pressure for solvent annealing, and the initial film thickness. A unique HPL with well-defined perforation in hexagonal lattice packing can be successfully formed, giving the feasibility to fabricate the SiO_2_ monolith with a hexagonal hole array after RIE treatment. Additionally, well-aligned parallel cylindrical nanostructure and an array of spherical microdomains can be induced by controlling the solvent annealing conditions, which give rise to the formation of the SiO_2_ line and dot pattern after RIE treatment. With the combination of the variety of the self-assembled morphologies as shown above from solvent annealing of the PS-*b*-PDMS thin film, our results clearly demonstrated the possibility of fabricating nanopatterns by aligning the second layer with the first layer to form hole-on-dot and line-on-hole nanopatterns with solvent annealing, followed by RIE treatment. Thus, by taking advantage of the sequential self-assembly, it is feasible to create 3D nanopatterns from single-composition PS-*b*-PDMS through solvent annealing *via* the layer-by-layer approach, giving the standard procedure as a proof-of-concept approach for 3D nanopatterning. Our proposed strategy could find applications for BCP-based nano-MEMS manufacturing that will be helpful in areas such as photovoltaics, batteries, and membranes, besides the demands from wafer industries.

## Supporting Information Available

Theoretical framework: SCFT, figures of TEM micrograph, and 1D SAXS profile of PS-*b*-PDMS after solution casting (PDF)

## References

[ref1] Park C.; Yoon J.; Thomas E. L. Enabling nanotechnology with self assembled block copolymer patterns. Polymer 2003, 44, 6725–6760. 10.1016/j.polymer.2003.08.011.

[ref2] Park M.; Harrison C.; Chaikin P. M.; Register R. A.; Adamson D. H. Block Copolymer Lithography: Periodic Arrays of ∼10 ^11^ Holes in 1 Square Centimeter. Science 1997, 276, 1401–1404. 10.1126/science.276.5317.1401.

[ref3] Bates F. S.; Fredrickson G. H. Block copolymer thermodynamics: theory and experiment. Annu. Rev. Phys. Chem. 1990, 41, 525–557. 10.1146/annurev.pc.41.100190.002521. 20462355

[ref4] Jung Y. S.; Ross C. A. Orientation-controlled self-assembled nanolithography using a polystyrene- polydimethylsiloxane block copolymer. Nano Lett. 2007, 7, 2046–2050. 10.1021/nl070924l. 17570733

[ref5] Jackson E. A.; Hillmyer M. A. Nanoporous membranes derived from block copolymers: from drug delivery to water filtration. ACS Nano 2010, 4, 3548–3553. 10.1021/nn1014006. 20695511

[ref6] Lo K.-H.; Chen M.-C.; Ho R.-M.; Sung H.-W. Pore-filling nanoporous templates from degradable block copolymers for nanoscale drug delivery. ACS Nano 2009, 3, 2660–2666. 10.1021/nn900299z. 19697943

[ref7] Peinemann K.-V.; Abetz V.; Simon P. F. Asymmetric superstructure formed in a block copolymer via phase separation. Nat. Mater. 2007, 6, 992–996. 10.1038/nmat2038. 17982467

[ref8] Hsueh H.-Y.; Chen H.-Y.; She M.-S.; Chen C.-K.; Ho R.-M.; Gwo S.; Hasegawa H.; Thomas E. L. Inorganic gyroid with exceptionally low refractive index from block copolymer templating. Nano Lett. 2010, 10, 4994–5000. 10.1021/nl103104w. 21047065

[ref9] Lim H. S.; Lee J.-H.; Walish J. J.; Thomas E. L. Dynamic swelling of tunable full-color block copolymer photonic gels via counterion exchange. ACS Nano 2012, 6, 8933–8939. 10.1021/nn302949n. 23020142

[ref10] Hsueh H. Y.; Ling Y. C.; Wang H. F.; Chien L. Y. C.; Hung Y. C.; Thomas E. L.; Ho R. M. Shifting networks to achieve subgroup symmetry properties. Adv. Mater. 2014, 26, 3225–3229. 10.1002/adma.201305618. 24677175

[ref11] Kang Y.; Walish J. J.; Gorishnyy T.; Thomas E. L. Broad-wavelength-range chemically tunable block-copolymer photonic gels. Nat. Mater. 2007, 6, 957–960. 10.1038/nmat2032. 17952084

[ref12] Lo T.-Y.; Krishnan M. R.; Lu K.-Y.; Ho R.-M. Silicon-containing block copolymers for lithographic applications. Prog. Polym. Sci. 2018, 77, 19–68. 10.1016/j.progpolymsci.2017.10.002.

[ref13] Cavicchi K. A.; Berthiaume K. J.; Russell T. P. Solvent annealing thin films of poly(isoprene-b-lactide). Polymer 2005, 46, 11635–11639. 10.1016/j.polymer.2005.09.072.

[ref14] Sinturel C.; Vayer M.; Morris M.; Hillmyer M. A. Solvent Vapor Annealing of Block Polymer Thin Films. Macromolecules 2013, 46, 5399–5415. 10.1021/ma400735a.

[ref15] Gotrik K. W.; Hannon A. F.; Son J. G.; Keller B.; Alexander-Katz A.; Ross C. A. Morphology control in block copolymer films using mixed solvent vapors. ACS Nano 2012, 6, 8052–8059. 10.1021/nn302641z. 22928726

[ref16] Lo T. Y.; Chao C. C.; Ho R. M.; Georgopanos P.; Avgeropoulos A.; Thomas E. L. Phase transitions of polystyrene-b-poly (dimethylsiloxane) in solvents of varying selectivity. Macromolecules 2013, 46, 7513–7524. 10.1021/ma4013863.

[ref17] Chang C. Y.; Manesi G. M.; Yang C. Y.; Hung Y. C.; Yang K. C.; Chiu P. T.; Avgeropoulos A.; Ho R. M. Mesoscale networks and corresponding transitions from self-assembly of block copolymers. Proc. Natl. Acad. Sci. U.S.A. 2021, 118, e2022275118 10.1073/pnas.2022275118. 33688050 PMC7980440

[ref18] Matsen M. W. Thin films of block copolymer. J. Chem. Phys. 1997, 106, 7781–7791. 10.1063/1.473778.

[ref19] Knoll A.; Horvat A.; Lyakhova K. S.; Krausch G.; Sevink G. J. A.; Zvelindovsky A. V.; Magerle R. Phase Behavior in Thin Films of Cylinder-Forming Block Copolymers. Phys. Rev. Lett. 2002, 89, 035501 10.1103/PhysRevLett.89.035501. 12144400

[ref20] Xiang J.; Lu W.; Hu Y. J.; Wu Y.; Yan H.; Lieber C. M. Ge/Si nanowire heterostructures as high-performance field-effect transistors. Nature 2006, 441, 489–493. 10.1038/nature04796. 16724062

[ref21] Park H. G.; Barrelet C. J.; Wu Y. N.; Tian B. Z.; Qian F.; Lieber C. M. A wavelength-selective photonic-crystal waveguide coupled to a nanowire light source. Nat. Photonics 2008, 2, 622–626. 10.1038/nphoton.2008.180.

[ref22] Stein G. E.; Kramer E. J.; Li X. F.; Wang J. Layering transitions in thin films of spherical-domain block copolymers. Macromolecules 2007, 40, 2453–2460. 10.1021/ma0625509.

[ref23] Tavakkoli K. G. A.; Gotrik K. W.; Hannon A. F.; Alexander-Katz A.; Ross C. A.; Berggren K. K. Templating three-dimensional self-assembled structures in bilayer block copolymer films. Science 2012, 336, 1294–1298. 10.1126/science.1218437. 22679094

[ref24] Ruiz R.; Sandstrom R. L.; Black C. T. Induced orientational order in symmetric diblock copolymer thin films. Adv. Mater. 2007, 19, 587–591. 10.1002/adma.200600287.

[ref25] Kim E.; Shin C.; Ahn H.; Ryu D. Y.; Bang J.; Hawker C. J.; Russell T. P. Size control and registration of nano-structured thin films by cross-linkable units. Soft Matter 2008, 4, 475–479. 10.1039/b717903k. 32907208

[ref26] Jung H.; Hwang D.; Kim E.; Kim B. J.; Lee W. B.; Poelma J. E.; Kim J.; Hawker C. J.; Huh J.; Ryu D. Y.; Bang J. Three-dimensional multilayered nanostructures with controlled orientation of microdomains from cross-linkable block copolymers. ACS Nano 2011, 5, 6164–6173. 10.1021/nn2006943. 21749153

[ref27] Kim S. Y.; Nunns A.; Gwyther J.; Davis R. L.; Manners I.; Chaikin P. M.; Register R. A. Large-area nanosquare arrays from shear-aligned block copolymer thin films. Nano Lett. 2014, 14, 5698–5705. 10.1021/nl502416b. 25211306

[ref28] Son J. G.; Hannon A. F.; Gotrik K. W.; Alexander-Katz A.; Ross C. A. Hierarchical nanostructures by sequential self-assembly of styrene-dimethylsiloxane block copolymers of different periods. Adv. Mater. 2011, 23, 634–639. 10.1002/adma.201002999. 21274911

[ref29] Shin D. O.; Mun J. H.; Hwang G. T.; Yoon J. M.; Kim J. Y.; Yun J. M.; Yang Y. B.; Oh Y.; Lee J. Y.; Shin J.; Lee K. J.; et al. Multicomponent nanopatterns by directed block copolymer self-assembly. ACS Nano 2013, 7, 8899–8907. 10.1021/nn403379k. 24007296

[ref30] Jin C.; Olsen B. C.; Luber E. J.; Buriak J. M. Preferential Alignment of Incommensurate Block Copolymer Dot Arrays Forming Moire Superstructures. ACS Nano 2017, 11, 3237–3246. 10.1021/acsnano.7b00322. 28225584

[ref31] Rahman A.; Majewski P. W.; Doerk G.; Black C. T.; Yager K. G. Non-native three-dimensional block copolymer morphologies. Nat. Commun. 2016, 7, 13988 10.1038/ncomms13988. 28004774 PMC5196037

[ref32] Liu R.; Huang H.; Sun Z.; Alexander-Katz A.; Ross C. A. Metallic nanomeshes fabricated by multimechanism directed self-assembly. ACS Nano 2021, 15, 16266–16276. 10.1021/acsnano.1c05315. 34647737

[ref33] Bai W.; Hannon A. F.; Gotrik K. W.; Choi H. K.; Aissou K.; Liontos G.; Ntetsikas K.; Alexander-Katz A.; Avgeropoulos A.; Ross C. A. Thin film morphologies of bulk-gyroid polystyrene-block-polydimethylsiloxane under solvent vapor annealing. Macromolecules 2014, 47, 6000–6008. 10.1021/ma501293n.

[ref34] Heitsch A. T.; Patel R. N.; Goodfellow B. W.; Smilgies D. M.; Korgel B. A. GISAXS characterization of order in hexagonal monolayers of FePt nanocrystals. J. Phys. Chem. C 2010, 114, 14427–14432. 10.1021/jp1047979.

[ref35] Nowak S. R.; Tiwale N.; Doerk G. S.; Nam C. Y.; Black C. T.; Yager K. G. Responsive blends of block copolymers stabilize the hexagonally perforated lamellae morphology. Soft Matter 2023, 19, 2594–2604. 10.1039/D3SM00142C. 36947412

[ref36] Smith A. P.; Douglas J. F.; Meredith J. C.; Amis E. J.; Karim A. Combinatorial study of surface pattern formation in thin block copolymer films. Phys. Rev. Lett. 2001, 87, 015503 10.1103/PhysRevLett.87.015503. 11461474

[ref37] Lo T. Y.; Dehghan A.; Georgopanos P.; Avgeropoulos A.; Shi A. C.; Ho R. M. Orienting block copolymer thin films via entropy. Macromolecules 2016, 49, 624–633. 10.1021/acs.macromol.5b02685.

[ref38] Kim H. C.; Park S. M.; Hinsberg W. D. Block copolymer based nanostructures: materials, processes, and applications to electronics. Chem. Rev. 2010, 110, 146–177. 10.1021/cr900159v. 19950962

[ref39] Li W.; Müller M. Directed self-assembly of block copolymers by chemical or topographical guiding patterns: Optimizing molecular architecture, thin-film properties, and kinetics. Prog. Polym. Sci. 2016, 54–55, 47–75. 10.1016/j.progpolymsci.2015.10.008.

[ref40] Metwalli E.; Krisch I.; Markovits I.; Rawolle M.; Ruderer M. A.; Guo S.; Wyrzgol S.; Jentys A.; Perlich J.; Lercher J. A.; Müller-Buschbaum P. Polymer-Coated PtCo Nanoparticles Deposited on Diblock Copolymer Templates: Chemical Selectivity versus Topographical Effects. ChemPhysChem 2014, 15, 2236–2239. 10.1002/cphc.201402047. 24838767

[ref41] Bita I.; Yang J. K.; Jung Y. S.; Ross C. A.; Thomas E. L.; Berggren K. K. Graphoepitaxy of self-assembled block copolymers on two-dimensional periodic patterned templates. Science 2008, 321, 939–943. 10.1126/science.1159352. 18703736

