# Supplementary material for: Sequential Self-Assembly of Polystyrene-block-Polydimethylsiloxane for 3D Nanopatterning via Solvent Annealing
Source: ACS Appl Mater Interfaces. 2024 Jul 22;16(30):40263–74. doi: 10.1021/acsami.4c08813 (PMC11299135; doi:10.1021/acsami.4c08813)
Supplement: Supplementary file 1 — am4c08813_si_001.pdf [file am4c08813_si_001.pdf]

Supporting Information for

Sequential Self-Assembly of Polystyrene-*block*-Polydimethylsiloxane for 3D Nanopatterning *via* Solvent Annealing

Thanmayee Shastry[1], Jiayu Xie[2], Cheng-Hsun Tung[1], Teoh Yen Lynn [1], Aum Sagar Panda[1], An-Chang Shi[2], and Rong-Ming Ho[1*]

[1]Department of Chemical Engineering, National Tsing Hua University, Hsinchu 30013, Taiwan

[2]Department of Physics & Astronomy, McMaster University, Hamilton, Ontario L8S 4M1, Canada

*Corresponding author: Rong-Ming Ho, Email: rmho@mx.nthu.edu.tw

Theoretical Framework: Self-Consistent Field Theory (SCFT)

The feasibility of achieving the hole-on-dot and line-on-hole patterns was investigated using Self-Consistent Field Theory (SCFT). In this section, we present a brief description of the SCFT. Here, we consider a system of n AB diblock copolymers confined in a thin-film geometry with a total volume V . Each diblock copolymer, modeled as a continuous Gaussian chain, contains a total of N segments, where $f_{\mathrm{A}}N(=fN)$ are segments of type A and $f_{\mathrm{B}}N(=(1-f)N)$ are segments of type B. For simplicity, we assume a uniform segment density ρ_0 so that the total volume of the copolymer melt is $V={}^{nN}/_{\rho_0}$. In our calculations, A is the minority block corresponding to the PDMS block while B is the majority block corresponding to the PS block. The confinement effect is incorporated by using the mask method [1-3]. In the mask method, an extra component is introduced into the system to act as walls that confine the system. The wall component is represented by a preassigned and fixed wall density, $\phi_{\mathrm{W}}(\boldsymbol{r})$.

After the standard mean-field treatment with the incorporation of the wall density, the Helmholtz free energy per chain has the form:

$$\frac{F}{nk_BT}=-\ln\mathrm{Q}-\frac{1}{V}\int dr\begin{bmatrix}N\omega_{\mathrm{A}}(\boldsymbol{r})\phi_{\mathrm{A}}(\boldsymbol{r})+N\omega_{\mathrm{B}}(\boldsymbol{r})\phi_{\mathrm{B}}(\boldsymbol{r})-\chi_{\mathrm{AB}}N\phi_{\mathrm{A}}(\boldsymbol{r})\phi_{\mathrm{B}}(\boldsymbol{r})\\-\chi_{\mathrm{AW}}N\phi_{\mathrm{A}}(\boldsymbol{r})\phi_{\mathrm{W}}(\boldsymbol{r})-\chi_{\mathrm{BW}}N\phi_{\mathrm{B}}(\boldsymbol{r})\phi_{\mathrm{W}}(\boldsymbol{r})\\+\eta(\boldsymbol{r})(1-\phi_{\mathrm{A}}(\boldsymbol{r})-\phi_{\mathrm{B}}(\boldsymbol{r})-\phi_{\mathrm{W}}(\boldsymbol{r}))\end{bmatrix}\quad(1)$$

Minimization of the above free energy with respect to the copolymer segment density and the conjugate fields results in a set of SCFT equations:

$$\begin{cases}N\omega_{\mathrm{A}}(\boldsymbol{r})=\chi_{\mathrm{AB}}N\phi_{\mathrm{B}}(\boldsymbol{r})+\chi_{\mathrm{AW}}N\phi_{\mathrm{W}}(\boldsymbol{r})+\eta(\boldsymbol{r})\\N\omega_{\mathrm{B}}(\boldsymbol{r})=\chi_{\mathrm{AB}}N\phi_{\mathrm{A}}(\boldsymbol{r})+\chi_{\mathrm{BW}}N\phi_{\mathrm{W}}(\boldsymbol{r})+\eta(\boldsymbol{r})\\\phi_{\mathrm{A}}(\boldsymbol{r})=\frac{1}{Q}\int_0^f q(s,\boldsymbol{r})q^{\dagger}(s,\boldsymbol{r})ds\\\phi_{\mathrm{B}}(\boldsymbol{r})=\frac{1}{Q}\int_f^1 q(s,\boldsymbol{r})q^{\dagger}(s,\boldsymbol{r})ds\\\phi_{\mathrm{A}}(\boldsymbol{r})-\phi_{\mathrm{B}}(\boldsymbol{r})-\phi_{\mathrm{W}}(\boldsymbol{r})=1\end{cases}\quad(2)$$

In Eqs. 2, the forward and backward propagators ($q(s,\boldsymbol{r})$ and $q^{\dagger}(s,\boldsymbol{r})$) describe the statistical mechanics of the polymer chains and satisfy the following modified diffusion equations:

$$\frac{\partial}{\partial s}q(s,\boldsymbol{r})=\nabla^2q(s,\boldsymbol{r})-\omega(s,\boldsymbol{r})q(s,\boldsymbol{r})\quad(3)$$

$$-\frac{\partial}{\partial s}q^{\dagger}(s,\boldsymbol{r}) = \nabla^2 q^{\dagger}(s,\boldsymbol{r}) - \omega(s,\boldsymbol{r})q^{\dagger}(s,\boldsymbol{r}) \tag{4}$$

where $\omega(s,\boldsymbol{r}) = \omega_{\mathrm{A}}(\boldsymbol{r})$ when $s \in [0,f]$ and $\omega(s,\boldsymbol{r}) = \omega_{\mathrm{B}}(\boldsymbol{r})$ when $s \in [f,1]$. We solve Eqs. 3 and 4 by using the pseudo-spectral method [4] with the initial conditions $q(0,\boldsymbol{r}) = 1$ and $q^{\dagger}(1,\boldsymbol{r}) = 1$.

To describe the interface between the polymeric and wall materials, a common choice of the form for the wall density is the hyperbolic tangent function [5, 6]. To reflect the geometries of the PS-coated PS-*b*-PDMS thin film in the first layer, wall densities with desired morphologies need to be constructed. To achieve this, we first set up a $\phi_{\mathrm{W}}(\boldsymbol{r}) = 0.5$ surface (wall surface) that separates the polymer-rich region (Ω_{P}) and wall-rich region (Ω_{W}) in the 3-dimensional space. The desired geometry is depicted by the shape of the wall surface. We then assign values to $\phi_{\mathrm{W}}(\boldsymbol{r})$ at all spatial points in the computational box, which contains one unit cell of the periodic nanostructure. Specifically, for each spatial point $\boldsymbol{r}$, we determine the shortest distance between that point and the wall surface, denoted as r_N, and assign $\phi_{\mathrm{W}}(\boldsymbol{r})$ according to

$$\phi_{\mathrm{W}}(\boldsymbol{r}) = \begin{cases} \frac{1}{2}\left[1 - \tanh\left(\frac{r_N}{\lambda}\right)\right], \text{ if } \boldsymbol{r} \in \Omega_{\mathrm{P}} \text{ and } r_N < \sigma \\ 0, \text{ if } \boldsymbol{r} \in \Omega_{\mathrm{P}} \text{ and } r_N \geq \sigma \\ \frac{1}{2}\left[1 - \tanh\left(\frac{-r_N}{\lambda}\right)\right], \text{ if } \boldsymbol{r} \in \Omega_{\mathrm{W}} \text{ and } r_N < \sigma \\ 1, \text{ if } \boldsymbol{r} \in \Omega_{\mathrm{W}} \text{ and } r_N \geq \sigma \end{cases} \tag{5}$$

Here λ describes the steepness of the polymer-wall interface and σ is a cut-off distance. Throughout this work, we keep $\lambda = 0.1R_g$ and $\sigma = 0.25R_g$constant, where R_g is the unperturbed radius of gyration of the AB diblock copolymer. Additionally, we set $\phi_{\mathrm{W}}(\boldsymbol{r}) = 1$ wherever $\phi_{\mathrm{W}}(\boldsymbol{r}) > 0.95$ to facilitate the convergence of the SCFT equations. The resulting $\phi_{\mathrm{W}}(\boldsymbol{r})$ as a function of r_N is plotted in **Figure S1**.

With a preassigned wall density, we initialize the polymer density and numerically solve the SCFT equations. We solve Eqs. S3 and S4 pseudospectrally using the second-order operator-splitting method and iterate Eq. S2 by simple mixing.[7] The chain contour is

discretized into 100 pieces and 64 grid points are used to discretize each spatial dimension. After solving the SCFT equations for different nanostructures, we can determine their relative stability by comparing their free energy per chain (Eq. S1). To confirm that the spatial resolution used in our calculations is sufficient, we repeated the calculations for a small number of selected phases using twice the number of grid points (128) along each spatial dimension. We observed that the relative free energies only shifted marginally, indicating that 64 grid points lead to sufficiently accurate results.

Wall-Profile Construction

To investigate the thermodynamic stability of the experimentally observed lines-on-holes and holes-on-dots patterns, we design two different wall profiles to mimic the holes and dots geometries of the first (bottom) layer. **Figures S2a** and **b** illustrate the wall geometries used to mimic holes and dots, respectively.

In our calculations, we use $\chi_{\mathrm{AB}}N = 30$. The bottom walls represent the PS coating layer, while the top walls represent the PS blocks of the wetting layer, making both the bottom and top walls selective to the PDMS (B) blocks. This selectivity is achieved by setting $\chi_{\mathrm{AW}}N = 30$ and $\chi_{\mathrm{BW}}N = 0$. The A-block compositions f_{A} used for the cases of the holes and dots geometries are 0.34 and 0.41, respectively, matching those of the samples used in experiments. To estimate the length scales between the dots or holes in the first layer, we first performed SCFT calculations confined between two flat walls to obtain the dots (hexagonally packed spheres) and holes (hexagonally perforated lamellae) structures (**Figure S3a**), in which the free energies of the different structures are also minimized with respect to L_x and L_y. This allows us to estimate the L_x and L_y to be used in the calculations for the second layer. We found that the optimized L_x for holes is $\sim 5R_g$ and that for dots is $\sim 4.5R_g$ (**Figure S3b**). The L_y for both cases is related to L_x *via* $L_y = \sqrt{3}L_x$. We note that although the optimized periodicities for holes and dots change slightly with variations in f_{A} and D, these values provide reasonable

estimations for L_x to be used in the top-layer calculations. For the other geometric parameters, *i.e.*, R and h, we set $\{R, h\} = \{Lx/3, R\}$ for the holes shown in **Figure S2a** and $\{R, h\} = \{Lx/3, R/2\}$ for the dots shown in **Figure S2b**. For both wall geometries, we perform calculations for all candidate structures spanning a range of D. The procedure to generate the candidate phases for each wall geometry, as well as the morphologies of the candidate phases (**Figures 9** and **10**), can be found in the main text.

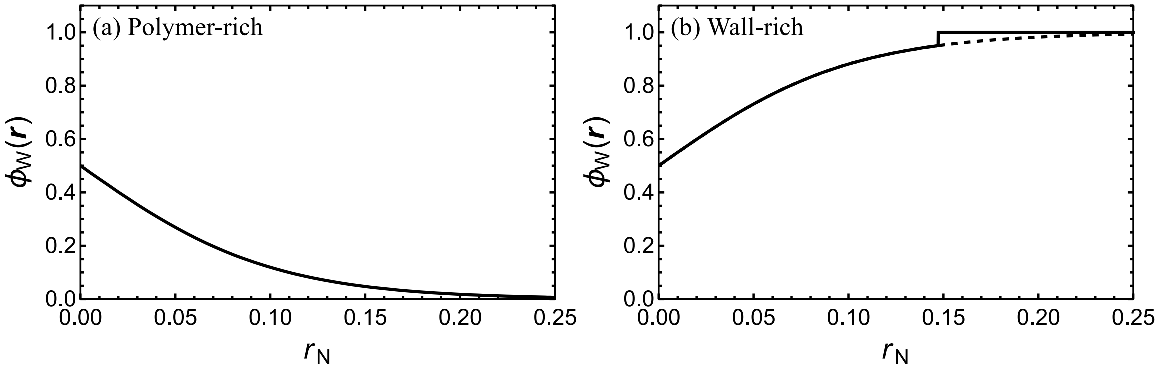

Figure S1. The wall density $\phi_W(\boldsymbol{r})$ as a function of r_N in the (a) polymer-rich and (b) wall-rich regions.

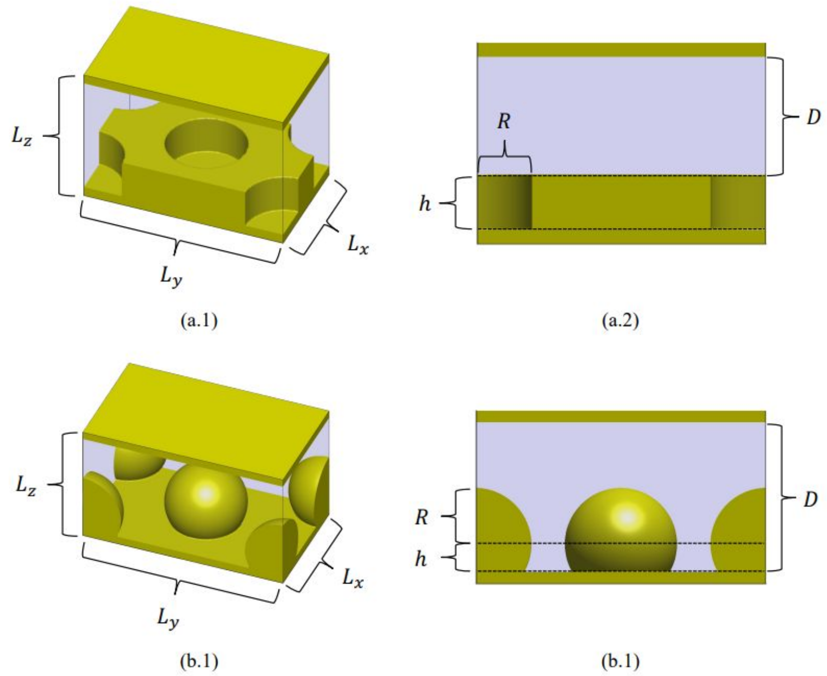

Figure S2. The schematics of the two wall geometries used in our SCFT calculation: (a) holes

and (b) dots. (α.1) and (α.2) with α=a or b provide two different views of the same simulation box. Wall-rich regions are colored yellow and polymer-rich regions are colored light blue (semi-transparent). Parameters characterizing different length scales are also marked.

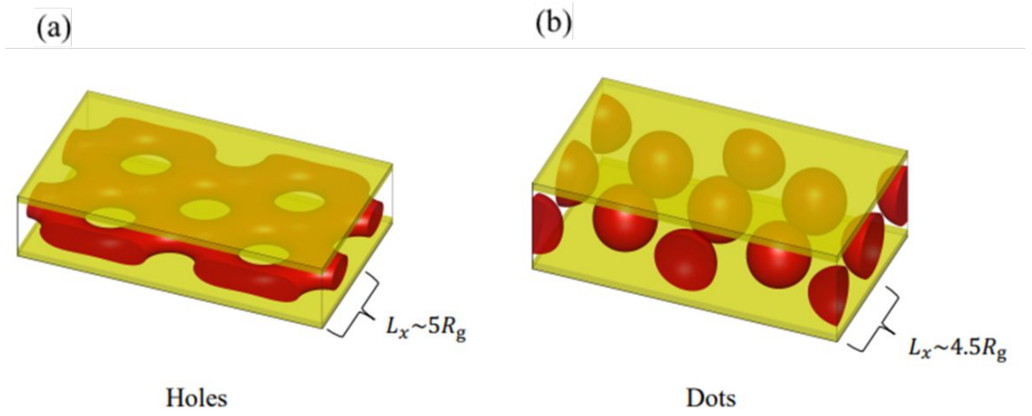

Figure S3. Schematics for the nanostructures in the first layer: (a) holes; (b) dots. The optimized L_x for each structure obtained from SCFT is also labeled.

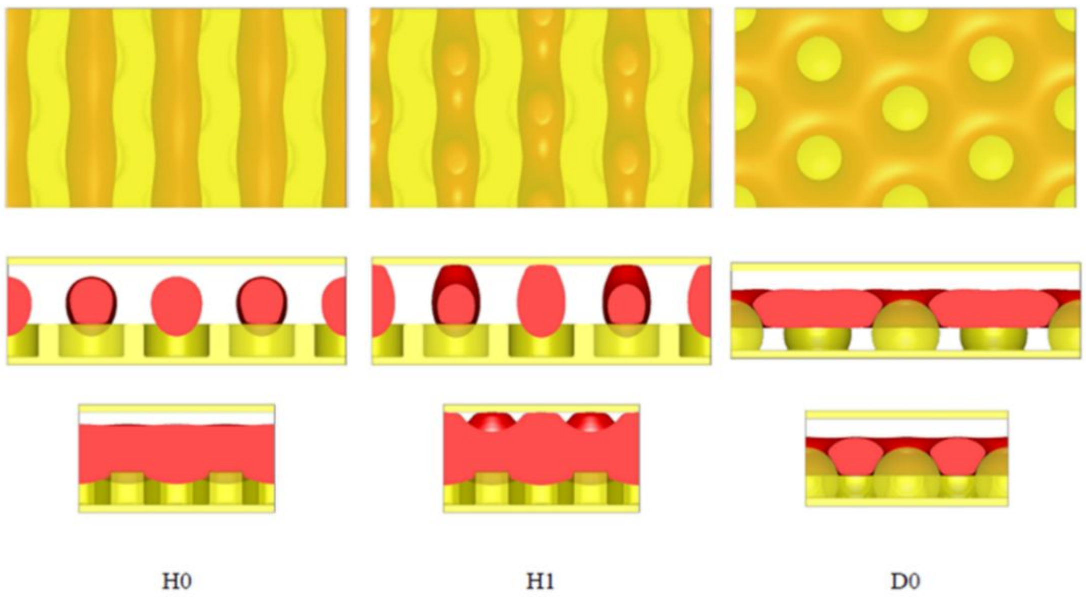

Figure S4. More detailed visualizations of the H0, H1, and D0 structures. The top, middle, and bottom rows are projections on the x−y, z−y, and z−x planes, respectively.

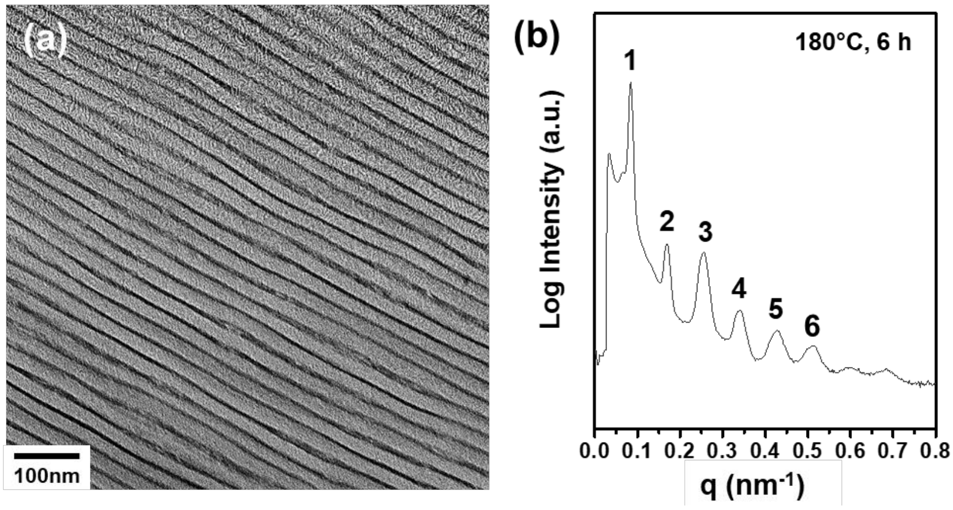

Figure S5. (a) TEM micrograph and (b) corresponding 1D SAXS profile of solution-cast PS-*b*-PDMS from cyclohexane (a neutral solvent) after thermal annealing at 180°C for 6 hours.

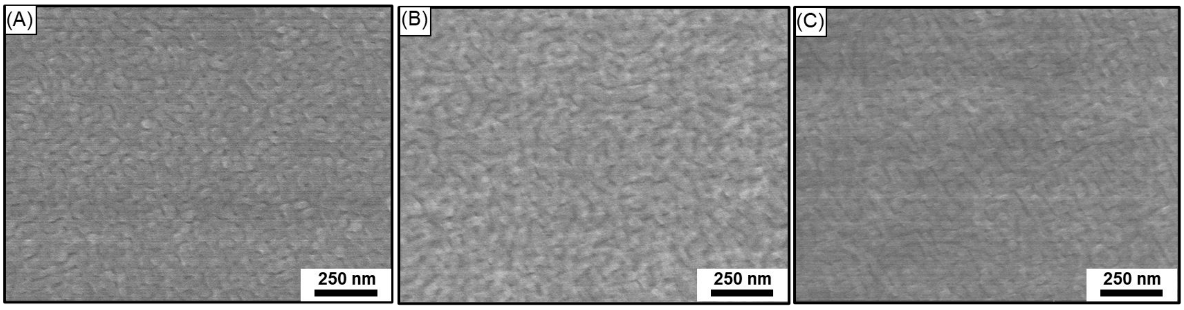

Figure S6. Top-view FESEM images of as-cast PS-*b*-PDMS thin films with initial thicknesses of (A) ~65 nm; (B) ~95 nm; (C) ~110 nm prior to the solvent annealing.

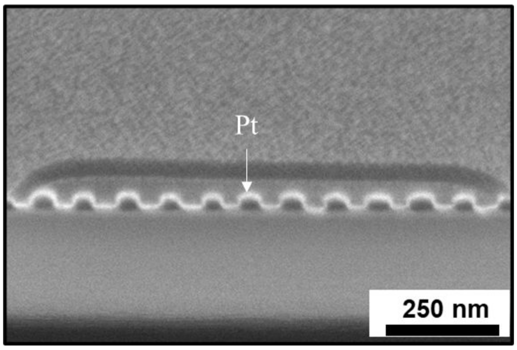

Figure S7. Cross-section view FESEM image after RIE treatments at which the sample was coated with Pt as contrast guiding line for the identification of forming hole arrays.

References

1. Matsen, M.W., Thin films of block copolymer. *The Journal of chemical physics* **1997,** *106*, 7781-7791.
2. Khanna, V.; Cochran, E.W.; Hexemer, A.; Stein, G.E.; Fredrickson, G.H.; Kramer, E.J., Li, X.; Wang, J.; Hahn, S.F., Effect of chain architecture and surface energies on the ordering behavior of lamellar and cylinder forming block copolymers. *Macromolecules* **2006,** *39*, 9346-9356.
3. Bosse, A.W.; Garcia-Cervera, C.J; Fredrickson, G.H., Microdomain ordering in laterally confined block copolymer thin films. *Macromolecules* **2007,** *40*, 9570-9581.
4. Shi, A.C., Self-consistent field theory of inhomogeneous polymeric systems. *Variational Methods in Molecular Modeling* **2017,** 155-180.
5. Yang, G.; Tang, P.; Yang, Y; Cabral, J.T., Self-assembly of AB diblock copolymers under confinement into topographically patterned surfaces. *The Journal of Physical Chemistry B* **2009,** *113*, 14052-14061.
6. Zhang, L.; Yang, J.; Li, W., Emergence of Multi-strand Helices from the Self-Assembly of AB-Type Multiblock Copolymer under Cylindrical Confinement. *Macromolecules* **2022,** *55*, 9334-9343.
7. Rasmussen, K.Ø.; Kalosakas, G., Improved numerical algorithm for exploring block copolymer mesophases. *Journal of Polymer Science Part B: Polymer Physics* **2002,** *40*, 1777-1783.
